# Calcifediol boosts efficacy of ChAdOx1 nCoV-19 vaccine by upregulating genes promoting memory T cell responses

**DOI:** 10.1038/s41541-024-00909-w

**Published:** 2024-06-20

**Authors:** Himanshu Singh Saroha, Swati Bhat, Liza Das, Pinaki Dutta, Michael F. Holick, Naresh Sachdeva, Raman Kumar Marwaha

**Affiliations:** 1 Department of Endocrinology, Post Graduate Institute of Medical Education and Research (PGIMER), Chandigarh, 160012 India; 2 Department of Section on Endocrinology, Diabetes, Nutrition & Weight Management, Department of Medicine, School of Medicine, Boston University, Boston, MA USA; 3 Department of Endocrinology, International Life Sciences Institute (ILSI) and Society for Endocrine Health Care of Elderly, Adolescents and Children (SEHEAC), New Delhi, India

**Keywords:** Outcomes research, Adjuvants

## Abstract

The ChAdOx1 nCoV-19 (COVISHIELD) vaccine has emerged as a pivotal tool in the global fight against the COVID-19 pandemic. In our previous study eligible subjects were supplemented with calcifediol, a direct precursor to the biologically active form of vitamin D, calcitriol with an objective to enhance the immunogenicity of the COVISHIELD vaccine. Herein we investigated the effects of calcifediol supplementation on gene expression profiles in individuals who received the COVISHIELD vaccine. Peripheral blood mononuclear cells were isolated from vaccinated individuals with and without calcifediol supplementation at baseline, 3rd and 6th month, and the gene expression profiles were analyzed using high-throughput sequencing. The results revealed distinct patterns of gene expression associated with calcifediol supplementation, suggesting potential molecular mechanisms underlying the beneficial effects of calcifediol in improving the efficacy of COVISHIELD vaccine via augmentation of T cell activation, proliferation and T cell memory responses. Additionally, there was upregulation of NOD like receptor, JAK/STAT and TGF beta signaling pathways. Calcifediol supplementation in vaccinated individuals also downregulated the pathways related to the Coronavirus disease. Taken together, our findings provide valuable insights into the interplay between vitamin D receptor (VDR) signaling and vaccine-induced immune responses and offer another approach in improving vaccination induced antiviral responses.

## Introduction

Vitamin D is a fat-soluble vitamin, which has both skeletal as well as extra skeletal benefits, holds a crucial place in maintaining an individual’s holistic health and wellness. It is synthesized by the non-enzymatic conversion of 7-dehydrocholesterol upon exposure of the inner layers of the epidermis to ultraviolet (UV-B) rays of wave-length 290–320 nm from the sun. Vitamin D undergoes a two-step hydroxylation process for conversion to its active form, the first hydroxylation, facilitated by 25-hydroxylase, occurs in the liver, resulting in 25-hydroxyvitamin D (calcifediol); the second hydroxylation, takes place in the kidneys and certain other tissues, yielding 1,25-dihydroxyvitamin D (Calcitriol) through the catalytic activity of enzyme 25(OH)D3-1α-hydroxylase which is encoded by the gene *CYP27B1* (cytochrome P450 family 27 subfamily B member 1)^1^. Upon binding of 1,25(OH)2D to its ligand, vitamin D receptor (VDR), the VDR associates with its heterodimeric partner, retinoid X receptor, and subsequently interacts with vitamin D response elements (VDREs) present in the target genes (Supplementary Fig. 1)^2^. Initially, vitamin D was well known for its role in regulating calcium homeostasis and promoting the development of strong and healthy bones. However, studies in past few years have demonstrated that benefits of vitamin D extend beyond bone health and include the maintenance of a healthy immune system as most of the immune cells express VDR and VDREs^3^. Vitamin D influences the differentiation and function of various immune cells, including T cells, B cells, dendritic cells, and macrophages. Vitamin D can suppress the production of pro-inflammatory cytokines, such as tumor necrosis factor-alpha (TNF-α) and interleukin-6 (IL-6), while increasing the expression of anti-inflammatory cytokines, such as interleukin-10 (IL-10), and promoting the differentiation of Th2 and Treg cells, which are important in maintaining immune tolerance^4,5^. Additionally, vitamin D enhances production of antimicrobial peptides that can kill viruses and bacteria via triggering genes downstream of VDR like cathelicidins, *CYP24*, and *DEFB4*^6^.

Due to erroneous beliefs that a normal diet and a few minutes in the sun are sufficient sources of vitamin D, dietary deficiencies are consistently underdiagnosed^3^. High altitudes, seasonal variations, avoidance or inadequate sun exposure, dark skin, aging, and excessive sunscreen use are the factors causing vitamin D insufficiency by reducing the amount of UV radiation that reaches the skin. Low levels of vitamin D have been linked to diseases like inflammatory bowel disease^7^ and a higher risk of respiratory infections, such as COVID-19, influenza as well as autoimmune diseases^8,9^. Various studies and meta-analysis during COVID-19 pandemic indeed found an association between low vitamin D levels and a higher risk of severe COVID-19 outcomes, such as hospitalization and death^10–13^. The proposition has been put forward that activation of VDR may have the capacity to decrease the likelihood of acute respiratory distress syndrome (ARDS), cardiac complications, coagulopathy, and mortality in patients with COVID-19^10–14^. Calcifediol, an immediate precursor of calcitriol, represents a potential option available for mitigating the adverse effects of vitamin D deficiency, including functioning of the immune system^15^. In comparison to cholecalciferol, calcifediol has a longer half-life, better absorption and is more effective in raising 25-hydroxyvitamin D levels^16,17^. Calcifediol can be administered orally as a dietary supplement, making it more convenient and accessible than other drugs that require injection or intravenous administration.

In a recent study, we conducted an open-label, placebo-controlled, interventional trial to investigate whether calcifediol (25(OH)D3) supplementation boosts the efficacy of the ChAdOx1 nCoV-19 vaccine. Herein we observed that ChAdOx1 nCoV-19 vaccine recipients receiving oral calcifediol for 6 months attained more circulating 1,25(OH)2D levels as compared to those on placebo. Calcifediol supplementation resulted in a decline in the cumulative percentage of probable COVID-19 disease in all the groups. Calcifediol supplementation also resulted in a higher in vitro proliferation of lymphocytes stimulated with SARS-CoV-2 S-peptide pool coupled with a higher IL-10 and TGF-β and decreased expression of IL-6. The results suggested that calcifediol supplementation possibly triggered memory T cell responses and promoted hybrid immunity along with a Treg or Th2 phenotype^18^.

The present study was planned to unravel how calcifediol supplementation influences the gene expression profile of immune cells in vaccinated individuals. Our rationale was to understand the crosstalk between the VDR signaling and vaccine-mediated immune responses. Therefore, we further isolated the peripheral blood mononuclear cells (PBMCs) of the representative subjects and performed RNA-seq analysis to distinguish variations in gene expression between subjects who received calcifediol versus those on placebo (control group), at three distinct time points: baseline, 3rd month, and 6th month. To the best of our knowledge, there is no earlier study on the role of Calcifediol or Vitamin D supplementation in COVID vaccine recipients, analyzing changes at gene level in the immune cells. The results of RNA-seq analysis provided valuable insights into the differentially regulated genes and molecular pathways regulated by vitamin D and the potential pathways involved in improving the efficacy of COVISHIELD vaccine and augmentation of genes associated with innate and adaptive immune responses in conferring protection from COVID-19.

## Results

### Influence of Calcifediol supplementation on plasma 25(OH)D, iPTH, calcitriol, and serum calcium levels

**Fig. 1:**
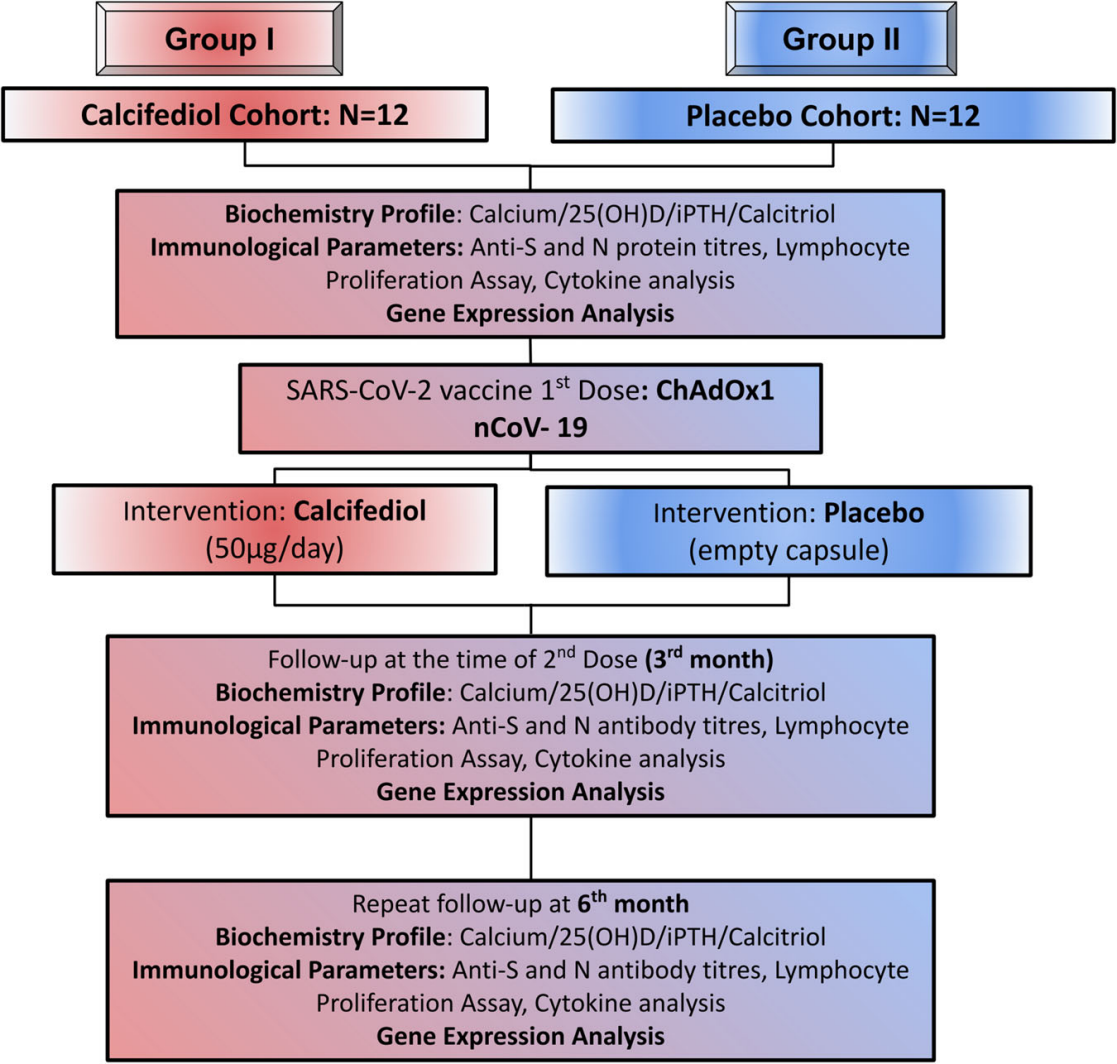
Study design. Flow diagram showing the number of subjects recruited in the calcifediol cohort (treated group) and the placebo cohort (placebo group) receiving intervention for 6 months, starting from the day of administration of the 1^st^ dose of ChAdOx1 nCoV-19 (COVISHIELD) vaccine. Various biochemical and immunological parameters were investigated at baseline, 3rd month, and 6th month. For gene expression analysis, peripheral blood mononuclear cells (PBMCs) were isolated and analyzed at baseline, 3rd month, and 6th month.

Participants were recruited to assess the impact of calcifediol supplementation on the efficacy of ChAdOx1 nCoV-19 vaccine. None of the subjects had COVID-19 disease prior to vaccination (Supplementary Table 1). The subjects were divided into 2 groups, the calcifediol arm and the placebo arm. After consideration of the criteria for inclusion and exclusion, a total of 24 adult subjects were selected and the transcriptomic analysis was performed at 3 time points; baseline, 3rd month, and 6th month as shown in the flow diagram (Fig. 1). Plasma levels of 25(OH)D, calcitriol, iPTH, and serum calcium at all time points were recorded (Table 1). Based on the questionnaire records and history obtained during each visit, all the subjects had similar duration of sun exposure/day. It may also be noted that anti-S antibody titers did not show any significant difference between the two groups, either at 3rd or 6^th^ month (Supplementary Fig. 2).

**Table 1:** Clinical and Biochemical characteristics of the study subjects at all time points

	Baseline			3rd Month			6th Month		
Characteristic	Placebo (*N* = 12)	Calcifediol (*N* = 12)	*p* value	Placebo (*N* = 12)	Calcifediol (*N* = 12)	*p* value	Placebo (*N* = 12)	Calcifediol (*N* = 12)	*p* value
Sex (female)	4	6	–	–	–	–	–	–	–
Age (years)	28.2 ± 9.1	35.8 ± 10	–	–	–	–	–	–	–
25(OH)D (ng/ml)	13.91 ± 5.98	11.39 ± 6.9	0.72	15.71 ± 6.95	134.9 ± 42.8	<0.0001	13.4 ± 5.94	85.01 ± 34.49	<0.0001
Δ t-baseline 25(OH)D	–	–	–	1.79 ± 5.73	123.53 ± 38	<0.0001	−0.51 ± 9.65	73.62 ± 31.8	<0.0001
Calcitriol (pmol/l)	134.84 ± 34.1	113.5 ± 32	0.34	132.14 ± 43.38	165.04 ± 53.24	0.29	120.5 ± 47.2	155.6 ± 50.6	0.25
Δ t-baseline Calcitriol	–	–	–	−2.7 ± 59.6	51.54 ± 49.78	0.03	−14.31 ± 49.71	42.14 ± 48.35	0.02
iPTH (pg/ml)	60.34 ± 28.67	53 ± 19.97	0.85	65.65 ± 37.08	38.1 ± 13.3	0.04	59.72 ± 26.16	52.5 ± 12.1	0.78
Δ t-baseline iPTH	–	–	–	5.3 ± 21.47	−18.47 ± 19.31	0.01	−0.61 ± 19.57	−0.45 ± 16.93	0.99
Calcium (mg/dl)	8.89 ± .08	9.37 ± 0.3	0.22	9.39 ± 0.5	9.55 ± 0.5	0.83	9.38 ± 0.6	9.61 ± 0.3	0.55
Δ t-baseline Calcium	–	–	–	0.5 ± 0.92	0.17 ± 0.4	0.52	0.48 ± 1.14	0.23 ± 0.4	0.68

The 25(OH)D, intact parathyroid hormone (iPTH) and calcitriol levels were measured in plasma while the calcium levels were determined in serum specimens. Δ t-baseline: Values of 3rd and 6th month with respect to baseline, where “t = 3rd or 6th month.” The data is shown as mean ± standard deviation (SD).

**Fig. 2:**
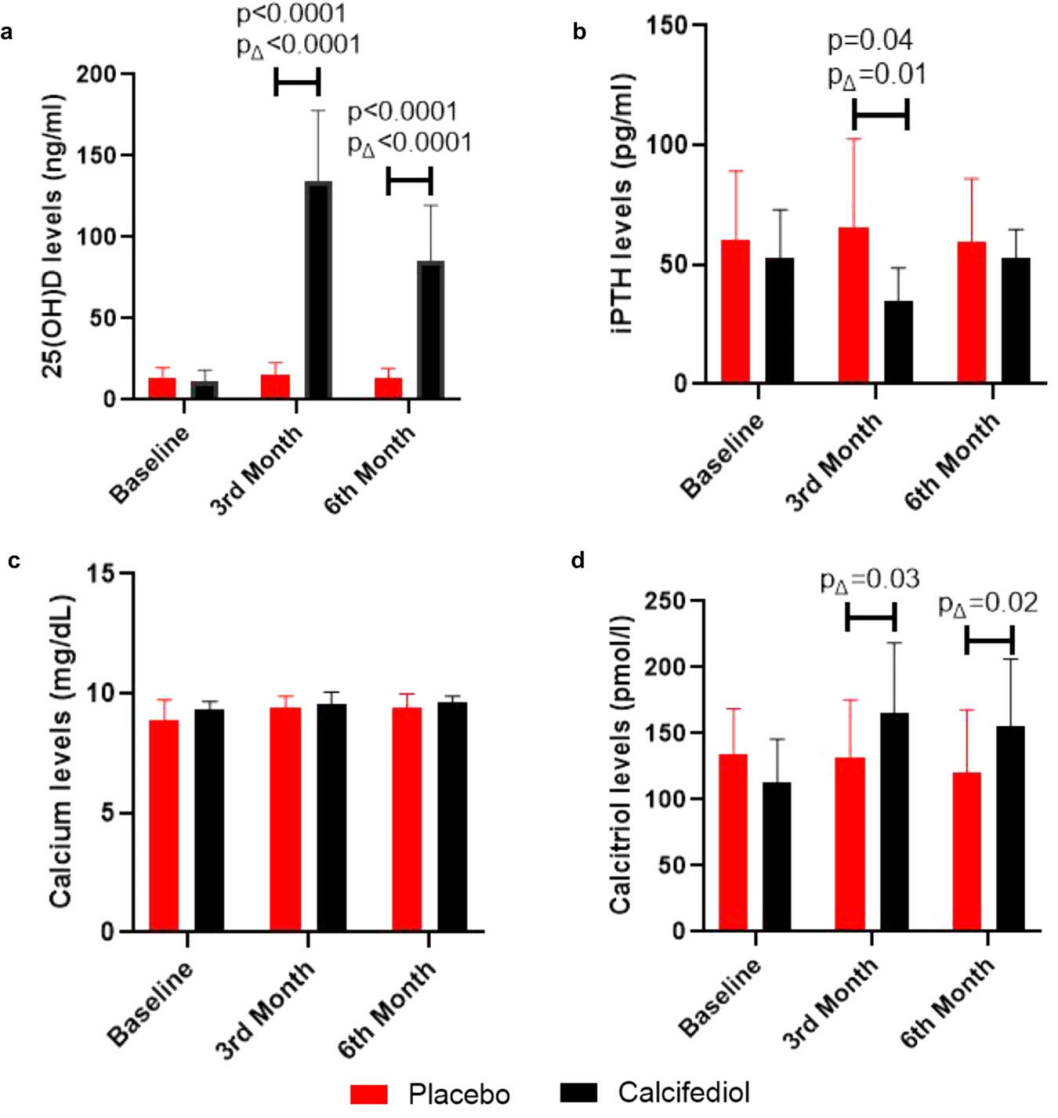
Impact of Calcifediol Supplementation on Calcemic Profile. Total plasma 25(OH)D, plasma iPTH, serum calcium and plasma calcitriol levels at baseline, 3rd month, and 6th month in both treated (*N* = 12) and placebo groups (*N* = 12). Here, “pΔ” represents *p* value of the significant delta changes between placebo and calcifediol supplemented groups. Δ t-baseline, represents change in values of 3rd and 6th month with respect to baseline. Two-way ANOVA was performed to determine the significance. The data is represented as Mean ± SD.

At first, we assessed the impact of calcifediol supplementation on plasma 25(OH)D levels. Baseline plasma 25(OH)D levels were similar in both the treated (mean ± SD, ng/ml) (11.39 ± 6.9) and the placebo cohort (13.91 ± 5.98) (*p* = 0.72). In the treated group, the plasma 25(OH)D levels (mean ± SD, ng/ml) increased at the 1st (73.20 ± 46.96) (*p* = 0.0006) (Supplementary Table 2), 3rd month (134.9 ± 42.8) (*p* < 0.0001) and then plateaued at the 6th month (85.01 ± 34.49) (*p* < 0.0001) post calcifediol supplementation, indicative of the effectiveness of calcifediol in boosting Vitamin D levels. In the placebo arm, the plasma 25(OH)D levels remained lower throughout the study period (Fig. 2a).

Elevated plasma iPTH levels are indicative of calcium deficiency, triggering processes to increase blood calcium, often at the expense of bone mineralization. Calcifediol supplementation may contribute in maintaining adequate calcium levels, potentially reducing the need for heightened iPTH secretion. At baseline, the plasma iPTH levels (mean ± SD, ng/ml) in the treated group (53 ± 19.97) were similar to the placebo cohort (60.34 ± 28.67) (*p* = 0.85). Post calcifediol supplementation, the iPTH levels in the calcifediol cohort dipped (38.1 ± 13.3 ng/ml) with a slight increase in the placebo cohort (65.65 ± 37.08 ng/ml) at 3rd month. At 6th month, there was no significant change in the plasma iPTH levels in both the groups (Fig. 2b).

Similarly, in the treated group, calcifediol supplementation did not alter the serum calcium levels (mg/dl) (9.55 ± 0.5) at 3rd month and (9.61 ± 0.3) level at 6th month (Fig. 2c). Nevertheless, this finding does not undermine the importance of vitamin D in maintaining optimal calcium levels for overall health, bone density, and proper immune function.

### Calcitriol concentrations and vitamin D signaling

Calcitriol is the active hormonal form of vitamin D, and plays a pivotal role in regulating calcium and phosphorus levels, bone mineralization, and immune responses. Individuals supplemented with calcifediol demonstrated elevated 25(OH)D levels and exhibited a significant increase in mean (±SD) calcitriol levels (pmol/l) at 3rd month (165.04 ± 53.24, Δ *p* = 0.03) and at 6th month (155.6 ± 50.6, Δ *p* = 0.02) (Table 1, Fig. 2d) when the delta increase in calcitriol levels was compared with baseline levels. Further, there was a positive correlation between 25(OH)D and calcitriol levels (r = 0.286, R^2^ = 0.082) (*p* = 0.0149) (Supplementary Fig. 3). This relationship underscores the importance of calcifediol as a precursor to calcitriol, emphasizing the superiority of calcifediol over native vitamin D and the clinical relevance of maintaining optimal vitamin D status.

### Outcome of calcifediol supplementation on the gene expression profile of PBMCs

At first, the proportion of lymphocytes and monocytes in the PBMCs were similar in the treated and placebo cohorts at baseline and subsequent time points.

**Fig. 3:**
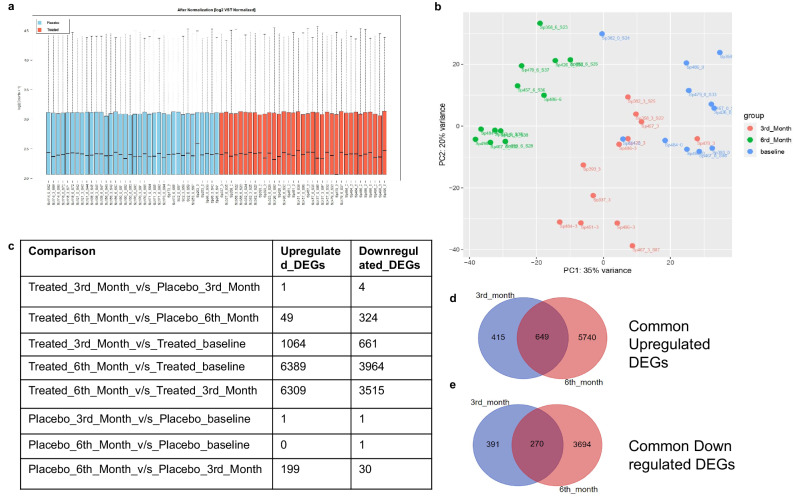
Differentially expressed genes (DEGs) identification. **a** Normalization of raw counts by DESeq 2 using Variance Stabilizing Transformation (VST). **b** Principal Component Analysis (PCA) of calcifediol supplemented (treated) cohorts, where blue bubbles represent baseline, red bubbles represent 3rd month and green bubbles represent 6th month. **c** Comparisons of the numbers of differentially expressed genes [differentially expressed (DE) features] at various time points. **d** Venn Diagrams depicting common and unique Upregulated and (**e**) downregulated differentially expressed genes in the calcifediol supplemented subjects at the 3rd and 6th month.

The raw count data of the subjects was normalized with variance stabilizing transformation (VST) method of DESeq2 (Fig. 3a) and all standard statistical analysis were performed^19–21^. The combined Principal Component Analysis (PCA) plot of both the cohorts showed natural heterogeneity (Supplementary Fig. 4) because of various confounding variables like age, BMI and gender (Supplementary Figs. 5, 6, 7). Since the placebo subjects did not receive any intervention, the PCA plot of the placebo group (Supplementary Fig. 8) did not show any clustering but randomly arranged at different time points, whereas the PCA plot of calcifediol intervention cohort (Fig. 3b) showed different clusters at each time-point as an outcome of intervention, indicating a time-dependent effect of the calcifediol supplementation. Therefore, the changes in gene expression at different time points were mainly compared within the treated group while the placebo group served as a reference cohort.

A subgroup analysis of gene expression from treated baseline to treated 3rd and treated 6th month revealed a total number of 1725 genes and 10353 genes respectively being expressed differentially at log2 fold change of 1 with a significance level of *p* < 0.05 (Fig. 3c). Upon comparing the differentially expressed genes (DEGs) of this subgroup, we found that 649 genes (Fig. 3d) were consistently upregulated and 270 genes (Fig. 3e) were consistently downregulated at both 3rd and 6th month during calcifediol supplementation. Since the comparison of the treated group at 6th month versus baseline demonstrated the maximum number of DEGs and majority of those DEGs also appeared in the 3rd month, this comparison was chosen primarily for downstream analysis.

### Differentially expressed genes (DEG) analysis

**Fig. 4:**
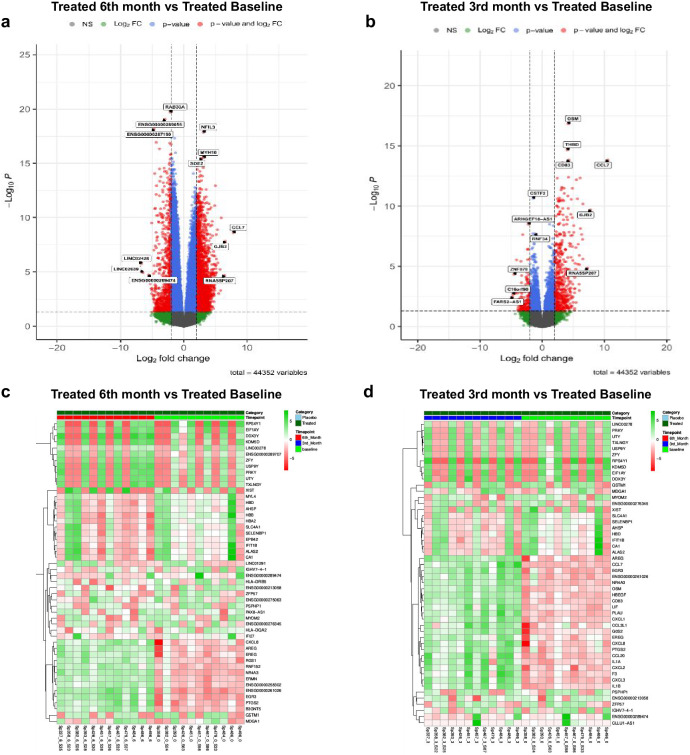
Differentially Expressed Genes (DEG) analysis. Volcano plots of differentially expressed genes (DEGs) between, **a** Treated 6th month vs Treated baseline, **b** Treated 3rd month vs Treated baseline. The log2 fold change was plotted against the *P* value (−10 log base 10). Significant DEGs genes with Log2 fold change > 1.0 and having adjusted *p* value < 0.05 are shown in red whereas the non-significant genes with Log2 fold change < 1.0 and having adjusted *p* value < 0.05 are shown in blue. Heatmaps of DEGs showing, **c** The top upregulated and downregulated genes of Treated 6th month vs Treated baseline and, **d** The top upregulated and downregulated genes of Treated 3rd month vs Treated baseline. The green color represents higher expression of genes and the red color denotes lower expression of genes and average expression is represented by white color.

DEG analysis of the calcifediol supplemented (treated) group at 6th month in comparison to baseline of the same group revealed that at the 6th month of calcifediol supplementation, a total of 10,353 genes were differentially regulated of which 6389 genes were upregulated and 3964 genes were downregulated. Whereas 1064 genes were found to be upregulated and 661 genes were downregulated at 3rd month as compared to baseline. As depicted in the respective volcano plots the significant differentially expressed genes with fold change more than 1 and having adjusted *p* value < 0.05 are marked in bright red whereas the non-significant genes with fold change less than 1 and having adjusted *p* value > 0.05 are marked in light blue (Figs. 4a, b) and the top 50 DEGs whose expression had most variance in these two sub-analyses were depicted in heatmaps (Fig. 4c, d) respectively. Genes involved in multiple biological processes appeared to be involved and the key genes of this comparison included *PTGS2, EREG, RSG1, AREG, NR4A3, EGR3, GSTM1, CYP27B1* and *CYP24A1*. As a proof of concept, we also checked the expression of genes related to VDR signaling (VDREs) and found that the expression of these 114 genes increased at 3rd month and plateaued at 6th month of the calcifediol supplementation (Supplementary Fig. 9, Supplementary Table 3). As important mediators of vitamin D action, the expression of *VDR*, 25-hydroxyvitamin D 1-alpha-hydroxylase (*CYP27B1*) and vitamin D3 24-hydroxylase *(CYP24A1)* was assessed in the calcifediol supplemented subjects. At first, we observed, that the expression of VDR increased at both time points from baseline expression levels (Log2 fold change at 3rd month: 0.74, 6th month: 0.67). The expression of *CYP27B1* was high at 3rd month which plateaued at 6th month (Log2 fold change at 3rd month: 0.9, 6th month: 0.244). Further, in the calcifediol supplemented subjects, there was a mild increase in the expression of *CYP24A1* at the 3rd month followed by a higher increase at 6th month (Log2 fold change: 3rd month: 0.06, 6th month: 1.12 from baseline). As already stated, that plasma levels of calcitriol plateaued by 6th month, which coincides with increase in *CYP24A1* expression during that period (Supplementary Fig. 10). In view of the higher vitamin-D and calcitriol levels attained in the supplemented subjects, the expression of vitamin D binding protein (*DBP*), was also assessed. There was an increase in *DBP* expression at 3rd month, which reverted to baseline levels at 6th month (Log2 fold change; 3rd month: 2.217, 6th month: 0).

It may also be noted that, some of the genes related to differences in sex also appeared in the comparison in a few subjects (Fig. 4), although they did not influence the subsequent pathway analysis.

### Upregulation of innate and adaptive immune signaling pathways upon calcifediol supplementation

**Fig. 5:**
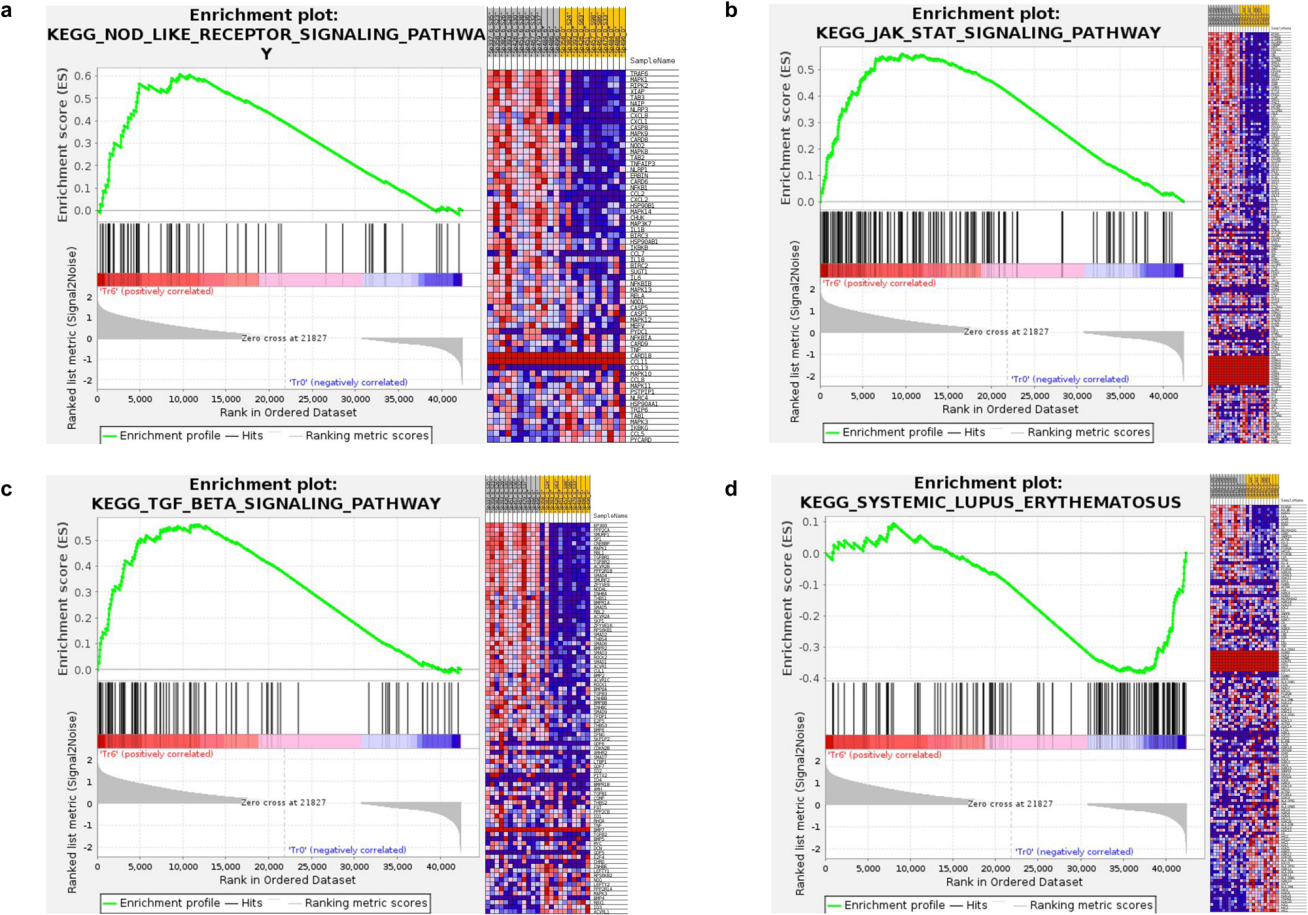
Gene Set Enrichment Analysis (GSEA). GSEA analysis of the KEGG pathway enrichment for the samples of Treated 6th month vs baseline revealed the genes involved in (**a**) NOD like receptor, (**b**) JAK/STAT and (**c**) TGF BETA signaling pathways that were upregulated and genes involved in (**d**) Systemic Lupus Erythematosus (SLE) that were downregulated in the same comparison. The ranked gene list is shown in the middle, red indicates upregulation, blue indicates downregulation and the black vertical line indicates the gene set.

The role of vitamin D in enhancing innate immunity is well known. When we compared the treated 6th month subjects versus their baseline data, the KEGG Gene set enrichment analysis (GSEA) revealed genes involved in the NOD like receptor, as well as JAK/STAT and TGF β signaling pathways, were upregulated at 6th month (Fig. 5), indicating higher induction of VDR signaling in DCs and macrophages potentially inducing antimicrobial or antiviral proteins like β-defensins and cathelicidins. While defensins protects the host cells by disrupting microbial membranes including SARS-CoV-2, cathelicidins attract various immune cells via chemotaxis and also enhance phagocytosis by macrophages, increasing vascular permeability and ultimately activation of B and proliferation of T cells^12^, thus strengthening innate immune responses that appear to be augmented by calcifediol supplementation.

In case of adaptive anti-viral responses, firstly, we observed a mild increase in the expression of *IFN-γ* at 3rd and 6th month post calcifediol supplementation in the treatment group (Log2 fold change; 3rd month: 0.149, 6th month: 0.44), which coincided with increase in its receptor, *IFNGR1* (Log2 fold change at 3rd month: 0.456, 6th month: 1.108). Key genes associated with T cell activation and proliferation including, *CD25, CD69, CD71* and *CD38* were found to be upregulated (*p* < 0.01 at 6th month). Genes associated with various anti-viral biological processes, including T cell receptor signaling pathway, anti-viral cellular responses and memory T cell responses were also upregulated (Table 2).

**Table 2:** List of main biological processes and the genes involved that were found differentially upregulated upon calcifediol supplementation

Biological Processes	Upregulated Genes
Activation of innate immune response	*PRKDC, SFPQ, MATR3, SIN3A*
Regulation of innate immune response	*XIAP, CASP8, LRP8, N4BP1, SAMHD1, APPL1, APPL2*
Positive regulation of T cell chemotaxis	*ADAM10, ADAM17, OXSR1, WNK1*
T cell receptor signaling pathway	*PDE4D, PIK3CA, PLCG2, PTPRJ, TRAF6, TXK, MALT1, PTPN22, RC3H2, WNK1, RC3H1, DENND1B*
Defense response to virus	*BCL2, LYST, DHX15, IFNAR2, NCBP1, EIF2AK2, RNASEL, PLA2G10, G3BP1, ZMYND11, CARD8, DDX58, SAMHD1, SETD2, TLR7, IFIH1, TRIM56, TRIM52, SLFN11, MLKL, PDE12, SERINC5, NLRP9, GBP7*
Cellular response to virus	*CHUK, MAPK14, DDX3X, FMR1, HIF1A, IL12A, LGALS8, RIOK3, IFIH1*
Cytokine-mediated signaling pathway	*CBL, IL6ST, JAK2, KIT, PTPN11, PTPRJ, SOS1, IRAK3, IL31RA*
Notch signaling pathway	*ADAM10, NOTCH2, MAML2*
B cell receptor signaling pathway	*BCL2, CD38, CTLA4, NFATC2, PLCG2, TEC, VAV3, BANK1, PLEKHA1*
Calcium ion transmembrane transport	*ATP2A2, ATP2B4, ITGAV, P2RX7, PKD2, RYR3, SLC8A1, TRPC1, TRPC5, SLC24A1, TRPM7, MCOLN3, TRPM6, ANO6, MCOLN2*
Memory T cell responses	*EOMES, ID3, ADAM23, EPHA4, ITK, BCL11B, PRDM1, CCR6, ZNF365, TGFBR3, DOCK9, INPP4B, EPHA4, ITK, BCL11B, PRDM1, PPP2R2B, SLFN5, FBXO32*

### Downregulation of coronavirus disease and inflammatory pathways

Upon comparing the treated 6th month with the treated baseline, the downregulated DEGs function was explored using KEGG pathway enrichment analysis. The enrichment analysis indicated that Coronavirus disease, Neutrophil extracellular trap formation (NET), Systemic lupus erythematosus (SLE), and Necroptosis were among the topmost down-regulated pathways. Notably, the Coronavirus disease pathway was one of the most consistently down-regulated pathways, not just in the comparison between the treated 6th month vs treated baseline (Supplementary Fig. 11) but also in other comparisons, mainly, treated 6th month vs treated 3rd month (Supplementary Fig. 12).

**Fig. 6:**
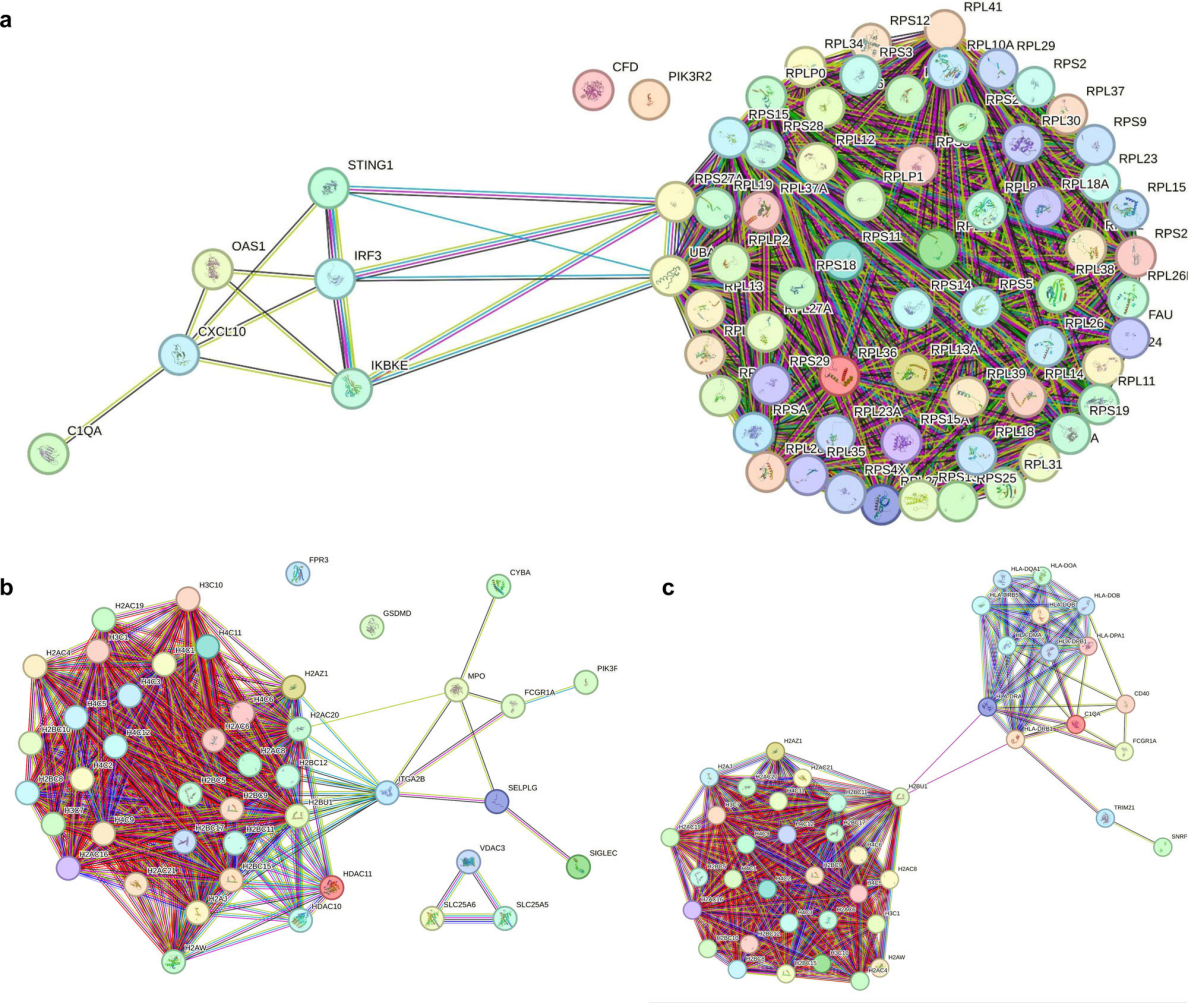
Protein-Protein Interaction (PPI) Networks. STRING analysis showing Protein-Protein Network of genes related to (**a**) Coronavirus Disease, (**b**) Neutrophil extracellular traps (NETs), and (**c**) Systemic lupus erythematosus (SLE) that are downregulated and showing significant Protein-protein interaction (PPI) enrichment (*P* < 1.0e-16). Network nodes represent proteins and each node represents all the proteins produced by a single, protein-coding gene locus. Edges represent protein-protein associations. These associations are meant to be specific and meaningful, i.e., proteins jointly contribute to a shared function; with or without physical binding with each other.

Next, the protein-protein interaction (PPI) network of the genes related to Coronavirus disease showed significant enrichment (*p* < 1.0e-16). Neutrophil extracellular trap formation (NET) and Systemic lupus erythematosus (SLE) also showed significant enrichment in PPI analysis (*p* < 1.0e-16) in the STRING database (Fig. 6). The upregulated pathways shown by KEGG pathway analysis in the treated 6th month vs treated baseline included JAK-STAT signaling, TGF beta signaling, NF-kappa B signaling pathway and cell cycle (Supplementary Fig. 13) some of which were also seen in comparison of treated 6th month versus 3rd month (Supplementary Fig. 14).

### Gene ontology analysis on the role of calcifediol supplementation in biological processes

The Gene ontology analysis revealed the role of calcifediol in upregulating about 376 biological processes (Supplementary Table 4) in comparison between treated 6th month versus treated baseline samples. Some of the relevant pathways included genes involved in the activation and regulation of innate immune responses, memory T cell responses, positive regulation of T cell chemotaxis signaling, T cell receptor signaling pathway, B cell receptor signaling, Notch signaling pathway, cytokine mediated signaling pathway, defense and cellular response to virus, and calcium ion transmembrane transport, many of which are important in generating antiviral responses. Of note, many genes that are related to generation and maintenance of memory T cells responses were found to be upregulated, indicating the positive impact of calcifediol supplementation in improving efficacy of COVISHIELD vaccine. The genes corresponding to these pathways are shown in Table 2. Calcifediol also downregulated nearly 157 biological processes (Supplementary Table 5). Some of these processes included mRNA splicing, via spliceosome, rRNA processing, and transcription by RNA polymerase II.

### Genes related to memory T cells are upregulated among the differential expressed genes

**Fig. 7:**
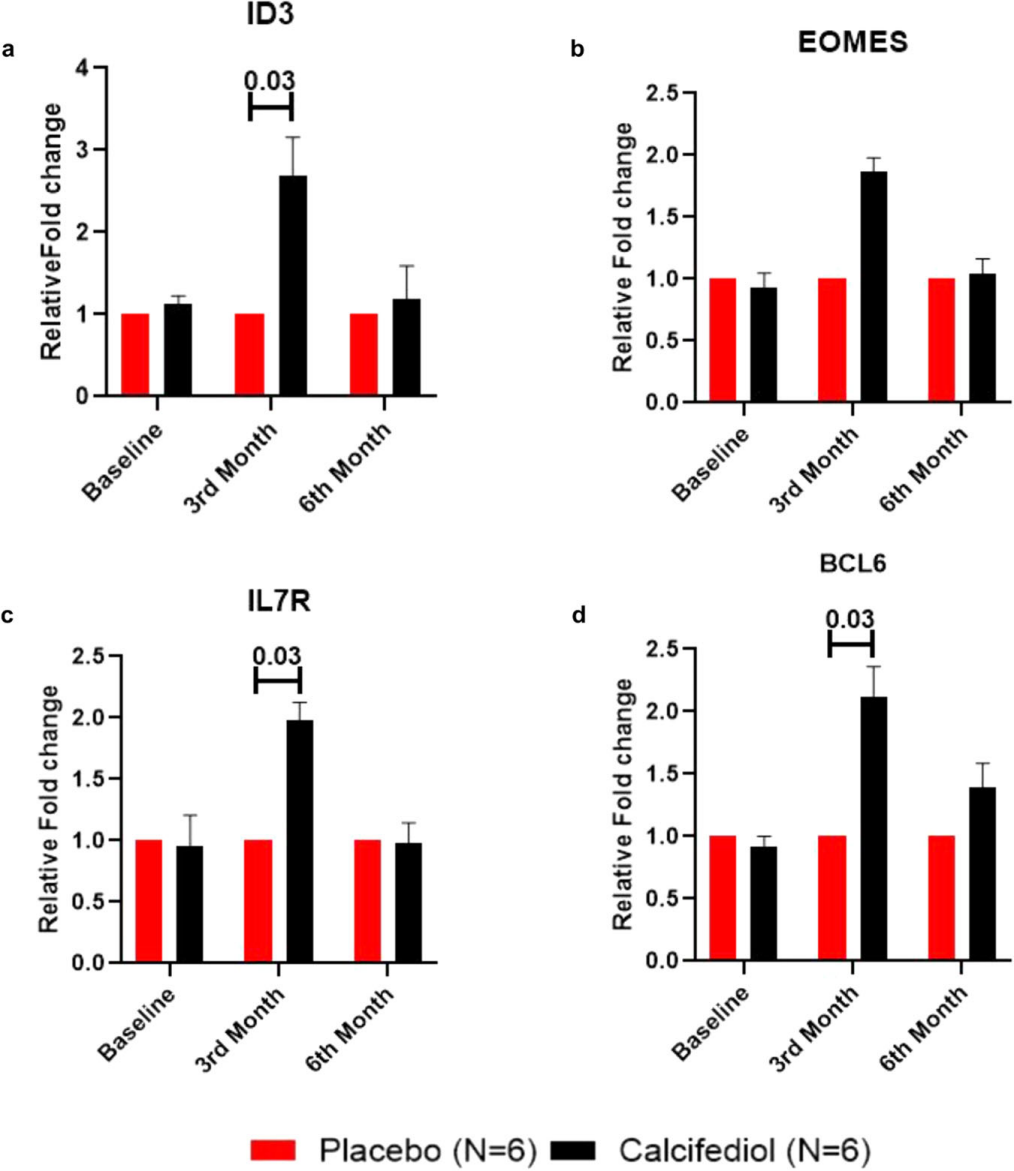
Activation of genes associated with memory T cell responses. Bar graphs show relative fold changes among placebo and treated subjects (*N* = 6) at baseline, 3rd month, and 6th month as validated by RT-PCR. Two-tailed student’s t-test were performed to determine the significance. The data is represented as Mean ± SD.

In the gene ontology analysis, many genes related to the memory T cell function were found to be upregulated. Since the main objective of the intervention (calcifediol) was to improve the efficacy of the COVISHIELD vaccine, therefore, we further tried to validate the expression of key genes required for the maintenance of memory T cell responses in representative samples. The expression of *EOMES, ID3, IL7R*, and *BCL6* was confirmed by RT-PCR using TaqMan probes (Supplementary Table 6) and the results were similar with RNA transcriptome analysis. We observed that the expression of all 4 genes, particularly, *ID3, IL7R*, and *BCL6* increased in 3rd month (*p* < 0.05) post calcifediol supplementation (Fig. 7). However, in the 6th month, the expression levels of these genes returned to near baseline levels. Taken together, the results from RT-PCR indicate improvement in memory T cell responses post-calcifediol supplementation in the subjects.

## Discussion

In our earlier study, we observed that calcifediol supplementation augmented protection from severe COVID-19 by promoting memory T cell responses and anti-inflammatory cytokines^18^. As mentioned previously, this was the first interventional trial with calcifediol supplementation in the recipients of ChAdOx1 nCoV-19 vaccine. The observed improvement in efficacy of COVISHIELD vaccination could be attributed to calcifediol supplementation but it can be scientifically proven by either immunophenotyping of SARS-CoV-2 specific T cells or by in vivo animal studies or by assessing the whole gene expression of immune cells of the recipients. Since VDR is expressed by almost all immune cells, we chose the latter approach and analyzed the gene expression profile of the treated and placebo subjects at regular time intervals. Moreover, hydroxylase activities especially those of 25(OH)D3-1α-hydroxylase are high in monocytes and macrophages and VDR signaling is active in lymphocytes^22,23^. Therefore, the current study undertook an important and critical question about the role of calcifediol on the gene expression profile in PBMCs of ChAdOx1 nCoV-19 vaccine recipients to understand the potential factors that can enhance the innate and adaptive immunity, improve memory T cell responses, reduce cytokine storm during viral exposure and ultimately boost vaccine efficacy.

In the gene expression analysis, the PCA plot of the treated group showed different clusters at each of the time points showing time dependent effect of the calcifediol supplementation as well as compliance of the subjects. The number of the upregulated genes were higher at the 6th month of supplementation than other comparisons, indicating long-term impact of the supplementation. As a proof of concept, we were able to observe increase in the expression of *VDR*, *CYP27B1* and VDREs in PBMCs which coincided with increase in calcitriol levels. Therefore, the observations could be attributed to the calcifediol-supplementation mediated activation of *VDR* and VDREs that act mainly via secondary messengers. Our major aim was to find out the genes associated with memory T cell responses; however, it must be noted that vitamin-D influences several pathways, therefore the results of DEG analysis showed genes involved in several biological processes. Earlier reports have indicated that vitamin D is involved both in the regulation of the innate immunity, as well as in the modulation of the adaptive immune response through direct effects on T cell activation and on the phenotype and function of antigen presenting cells^24,25^. Based on the high-throughput RNA sequencing data we have also found that vitamin D influences several pathways of innate as well as adaptive immune system such as the NOD like receptor, JAK/STAT and TGF beta signaling pathways. Most importantly, the pathway enrichment analysis revealed that Coronavirus disease was consistently downregulated in various comparisons including the treated 6th month vs treated baseline, treated 6th month vs treated 3rd month and even among treated 6th month and placebo 6th month. These findings further corroborate the clinical and immunological responses seen in subjects receiving calcifediol supplementation along with ChAdOx1 nCoV-19 vaccine^18^. Additionally, NETs and SLE were downregulated at the 3rd and 6th month post supplementation, clearly indicating that calcifediol had a direct impact on COVID-19 disease, inflammation and autoimmunity. Neutrophils are essential for innate immunity. However, COVID-19 disease, results in high levels of circulating NETs causing a reduction in blood flow in the capillaries leading to build-up of NETs in the lungs and other organs causing multiple organ failures^26^. Therefore, calcifediol appeared to modulate NETosis which might be important in curbing exaggerated inflammatory responses during the initial stages of secondary COVID infection. Vitamin D has also been reported in other diseases to reduce the formation of NETs^26,27^ including the treatment of SLE^28^.

One of the significant observations in our study was upregulation of several genes involved in the T cell activation and proliferation like *CD25, CD69, CD71*, and *CD38*, which is in line with our previous observation where calcifediol supplementation increased SARS-CoV-2 specific lymphocyte proliferation^18^. Likewise, the genes involved in the JAK-STAT pathway were also found upregulated which play a crucial role in augmenting cytokine responses in activated immune cells. In addition, upregulation of both subunits of the IFN-α receptor, a type of receptor involved primarily in antiviral defense was observed. In our previous study calcifediol has already been shown to increase serum IL-10 and the same is being reflected in this study as well. *IL17* was also downregulated which can be interpreted as ensuing tolerogenic milieu. Additionally, there was also upregulation of the suppressor of cytokine signaling (SOCS) family of genes like *SOCS1, 3, 4*, and *6* which are potent negative regulators of proinflammatory cytokine signaling via triggering a negative feedback loop on the JAK/STAT pathway, thus thwarting excess inflammation^29^. Another molecule associated with inhibitory immune signals, cytotoxic T lymphocyte‐associated protein 4 (*CTLA4*) was also found upregulated. CTLA4 is also known to be constitutively expressed in Tregs and is regarded as a key molecule for cell-mediated immunosuppressive functions^30^. CTLA-4 competitively binds to CD80/CD86 present on APCs, thereby impeding the binding of CD28 and thus subsequent prevention of secondary stimulus for T cell activation. Our data showed moderate upregulation of CTLA4 ligands (*CD80/CD86*) which highlighted the probable immune modulation by calcifediol. The zinc-finger transcription factor, *Helios*, which is critical for maintaining the anergic phenotype and suppressive activity of Tregs^31^ was upregulated reflecting that T cell mediated tolerogenic signals have been long delivered (at the time of infection or re-infection). *CD46* and *CD35* (CR1) that are expressed on activated T cells and lead to the development of Tregs in the presence of IL2^32^ were also seen upregulated. These findings highlight another arm of calcifediol-VDR signaling mediated regulation of adaptive immune responses that works in parallel to prevent exaggerated inflammatory responses during secondary COVID 19 infections.

In the gene ontology analysis various genes related to generation and maintenance of memory T cells responses were found to be upregulated. We further assessed a few genes that are expressed in lymphocytes and involved in the generation and maintenance of long-lived memory T cells. RT-PCR verified the genes associated with memory T cell responses including, *ID3, IL7R*, and *EOMES* showing a similar trend to transcriptome analysis. ID3hi memory T cells express higher levels of IL7R, enabling a greater responsiveness to IL7, and promoting memory T-cell survival and homeostasis^33,34^. Another gene, EOMES is required in function and homeostasis of effector and memory T cells^35^, indicating a transition of hyper-immune responses towards generation of memory T cells. *BCL*6 expression is transient and is required for the generation, but not maintenance, of memory CD8 + T cells^36^. The expression of most of these genes including *ID3, IL7R, EOMES*, and *BCL6* increased at 3rd month in the treated group which stabilized by the end of 6th month highlighting the role of vitamin D in promoting T cell memory.

There have been similar studies that have assessed the role of vitamin D in augmenting immunogenicity of different anti-COVID-19 vaccines with mixed outcomes (Supplementary table 7)^37–44^. Most of these studies used titers of anti-S antibodies as a measure of efficacy and the SARS-Cov-2 specific T cell responses including memory responses were not assessed. Therefore, in his context the findings of our study become more relevant, since despite of finding no changes in anti-S antibody titers, we could demonstrate improvement in efficacy of ChAdOx1 nCoV-19 vaccine and the data on gene expression of PBMCs supports the immunological findings of our index study. Recent trials on vitamin D supplementation, which showed improvement in the efficacy, mainly involving the mRNA vaccine (BNT162b2), measured outcomes around 2 months of supplementation^37–39^. This suggests that achieving vitamin D adequacy prior or during the vaccination period, yields maximum benefits. In contrast to these studies, we used calcifediol for supplementation and our subjects achieved higher levels of plasma vitamin D within one month that were maintained throughout the end of study.

The clinical implications of our findings extend to the realm of vaccine responses and overall immune health. A balanced calcium metabolism could create an environment conducive to optimal T cell activation, cytokine signaling, innate and adaptive immune responses, as highlighted in our gene expression analysis. Subjects receiving calcifediol sustained better calcium homeostasis, which in turn could positively influence immune responses. The mutuality observed between calcitriol, calcium and PTH levels during calcifediol supplementation underscored the potential clinical relevance of optimizing vitamin D status. Our observations on the impact of calcifediol supplementation on serum calcium levels were limited to a period of 6 months. However, similar studies in a larger number of cohorts might be useful in proving the superiority of calcifediol over vitamin D supplementation in facilitating calcium absorption and utilization, leading to better calcium homeostasis. From a clinical perspective, this could have implications for bone health, muscle function, and other calcium-dependent physiological functions. Taken together, the findings from gene expression analysis support a dual, pro as well anti-inflammatory role of vitamin D involving several factors of innate and adaptive immune signaling pathways. It can be inferred that owing to its immunomodulatory properties, vitamin D influences our immune system in a spatiotemporally controlled manner, by enhancing specific and regulated immunogenicity of SARS-CoV-2 vaccination in subjects with optimal vitamin D status thus, reducing the chances of immunological severity of SARS-CoV-2 infection caused by exaggerated inflammatory responses following infection or re-infection. Our results further necessitate the need to explore mechanisms by which calcifediol promotes memory T-cell survival and homeostasis at the molecular level.

There were many challenges associated with this study including the variable confounding factors like heterogeneity in subjects including genetic diversity, varying exposure to SARS-CoV-2 prior to intervention, diet, and lifestyle that were beyond our control and these factors ultimately make the analysis process challenging. Secondly, vitamin D toxicity could occur with plasma levels of 25(OH)D > 100 ng/ml, but none of our subjects presented with clinical or biochemical picture of vitamin D toxicity. However, renal ultrasonography and urinary calcium/creatinine ratio could have provided a better assessment of hypercalciuria in these subjects. We did not estimate serum DBP levels assuming that their levels are minimally influenced by levels of vitamin D or by its metabolites and none of these subjects were on estrogens, glucocorticoids or had any liver disorders^45^. The strengths included adequate sample size, compliance of subjects, and time points of the study. It may also be noted that since the study was conducted on a small population, the same conclusions cannot be generalized for a larger population that inherits different genetic backgrounds. Therefore, more studies are needed to identify the optimal doses and confirm the importance of calcifediol supplementation or maintaining adequate vitamin D levels.

To conclude, the study presents an efficacious approach to improve the efficacy of not just COVID-19 but other viral vaccines, which is highly safe and offers better calcium homeostasis important for various physiological functions.

## Methods

### Subject recruitment

The study was approved by the institutional ethics committee (IEC) of PGIMER (Post Graduate Institute of Medical Education & Research), Chandigarh, India. The recruited participants were healthy individuals aged 18–60 years, who had received two doses of the ChAdOx1 nCoV-19 vaccine (COVISHIELD ^TM^, Serum Institute of India, Pune, India) with a 12-week interval at the COVID-19 vaccination center of PGIMER. This was an open-label, placebo-controlled, interventional trial with two pre-specified groups: Calcifediol supplemented and a placebo group (CTRI/2021/08/035709). Of the 24 subjects who underwent screening, 12 received 50 µg calcifediol (2 capsules of 25 µg)/day (Dishman Carbogen Amcis Ltd, Ahmedabad, India) (Calcifediol-supplemented group), and the other 12 received placebo (2 capsules/day) (Placebo group). Both groups received the intervention for 6 months, starting from the first dose of the vaccine. All the subjects were advised to follow their normal lifestyle with no specific instructions for sun exposure. The team reinforced the importance of daily supplementation during each follow-up visit. Compliance was measured by collecting empty capsule bottles during each visit. Any negative reactions resulting from the use of calcifediol supplementation, including high levels of calcium in the blood or urine and renal calculi, were also noted. Blood samples were taken using three types of vials: plain (3 ml), EDTA (4 ml), and heparin (12 ml). The subjects were asked to return for follow-up appointments at 1, 3, and 6 months after receiving the vaccination. The SF-36 health survey questionnaire was administered to assess their quality of life at the start of the study and the final follow-up.

### Biochemical analyses

Serum calcium, inorganic phosphate, alkaline phosphatase, and albumin levels were measured to assess the calcemic profile using an automated biochemical analyzer (COBAS 8000, Roche Diagnostics, Germany). Electrochemiluminescence immunoassay (ECLIA) (COBAS e801, Roche Diagnostics, Germany) was used to measure plasma 25(OH)D (D represents either or both D2 and D3) and intact parathyroid hormone (iPTH) levels. Chemiluminescence immunoassay (CLIA, Diasorin Liason, Italy) was used to measure plasma 1,25(OH)2D levels. The anti-SARS-CoV-2 antibody titers were determined as described previously^18^.

### cDNA library preparation and RNA-sequencing

Total RNA was extracted from PBMCs by using the RNeasy Mini Kit (Qiagen, Germany) as per the manufacturer’s instructions. The concentration and integrity of total RNA were checked using the Qubit RNA Assay Kit in a Fluorometer (Qubit 4.0, Thermo Fisher Scientific, IN) while RNA quality was assessed with an automated nucleic acid electrophoresis system, Tapestation 4200^TM^ (Agilent Technologies, Santa Clara, USA). RNA samples were taken with an initial RNA input of 500 ng. “KAPA mRNA Capture kit” (KAPA Biosystems, CA, USA) was used for mRNA capture by oligo mag beads, followed by mRNA fragmentation using heat and magnesium. The KAPA RNA Hyper prep kit for Illumina sequencing was used for the construction of mRNA-seq libraries. Next, the cDNA was synthesized using a reverse transcriptase and random hexamers in a first-strand synthesis reaction. Subsequently, the cDNA was converted to double-stranded cDNA where Uracil is added instead of Thymine leading to the addition of dAMP to the 3′ ends of the resulting dsDNA. In adapter ligation, dsDNA adapters with 3′ dTMP overhangs were ligated to library insert fragments followed by library amplification, to amplify library fragments carrying appropriate adapter sequences at both ends using a high-fidelity, low-bias PCR. The library was sequenced using an Illumina NextSeq 2000 platform to generate 60 M, 2 × 150 bp reads/sample.

### Differentially expressed genes (DEGs) Identification

The raw data was evaluated for quality control using FastQC (version 0.11.9). Subsequently, Trim Galore (version 0.6.6) was utilized for the removal of poor-quality sequences and trimming of adapters. These processed reads were mapped to the reference transcriptome using the STAR (version 2.7.10a) alignment tool and then quantified using FeatureCounts (version 2.0.3), a module of the R package called R subread (version 2.0.1). For the annotation of Ensembl gene identifiers with gene symbols, descriptions, genomic locations, and biotypes, the R package BiomaRt (version 2.46.0) was employed to access the Ensembl database (version 103). To detect ribosomal RNA (rRNA) contamination, datasets were run against the SILVA database. Less than 1% of reads were annotated as rRNA which were filtered out from the datasets. The raw counts were normalized by DESeq 2 using Variance Stabilizing Transformation (VST). The count-based technology DESeq2 package (v.3.6.1) was operated in R (version 4.2.2) using Ubuntu (22.04.1 LTS) to conduct the differential expression analysis. Genes exhibiting a Log2 fold change at a cut off of one with Benjamini–Hochberg corrected p-value (false discovery rate, FDR) of less than 0.05 were considered significant vitamin D targets. Volcano plots and heatmaps were generated with the Enhanced Volcano (version 1.10.0) and Pheatmap (version 1.0.12) R packages respectively.

### Enrichment analyses and network visualization

Enrichment analysis and visualization of genes was performed using ‘Shiny GO (version 0.77)’ and ‘PathfindR (version 2.2.0)’ Gene Ontology (GO). GO enrichment analysis annotated the mechanism of action of target genes in three parts: biological process (BP), cellular composition (CC) and molecular function (MF). Enrichment analysis was performed to obtain bubble plots to annotate the upregulated and signaling pathways in which the target genes were involved, using the Kyoto Encyclopedia of Genes and Genomes (KEGG). For gene set enrichment analysis, GSEA (version 4.3.2 - build 13) was used to generate GSEA Plots. Protein-protein interaction networks were generated using STRING, a web-based tool.

### Statistical analysis

All standard statistical tools available in the DESeq2 package were used in the identification of DEGs. The output data was analyzed using Graphpad Prism (version 8.0). Normality of the data was checked by D’Agostino and Pearson normality test. Group analysis was performed by two-way ANOVA. The significance level was set at *p* < 0.05. The correlation was performed using Spearman correlation.

### Supplementary information

Supplemental Material

## Data Availability

The RNA-seq data supporting the findings of this study has been deposited in the Sequence Read Archive (SRA) database of NCBI, with the accession number PRJNA1113608, which can be accessed at the link, https://www.ncbi.nlm.nih.gov/sra/PRJNA1113608.
