# Supplementary material for: Calcifediol boosts efficacy of ChAdOx1 nCoV-19 vaccine by upregulating genes promoting memory T cell responses
Source: NPJ Vaccines. 2024 Jun 20;9:114. doi: 10.1038/s41541-024-00909-w (PMC11190216; doi:10.1038/s41541-024-00909-w)
Supplement: Supplementary file 1 — Supplemental Material [file 41541_2024_909_MOESM1_ESM.pdf]

SUPPLEMENTARY DATA

Calcifediol boosts the efficacy of ChAdOx1 nCoV-19 (COVISHIELD) vaccine via upregulation of key genes associated with memory T cell responses

[1]Himanshu Singh Saroha MSc, [1]Swati Bhat MTech, [1]Liza Das DM, [1]Pinaki Dutta DM, [2]Michael F. Holick PhD, [1]Naresh Sachdeva PhD[#], [3]Raman Kumar Marwaha DNB#

Departments of [1]Endocrinology, Post Graduate Institute of Medical Education and Research (PGIMER), Chandigarh 160012, India. [2]Section on Endocrinology, Diabetes, Nutrition & Weight Management, Department of Medicine, School of Medicine, Boston University, Boston, MA, USA. [3]Department of Endocrinology, International Life Sciences Institute (ILSI) and Society for Endocrine Health Care of Elderly, Adolescents and Children (SEHEAC), New Delhi, India.

Supplementary Figure 1: Vitamin D biosynthesis

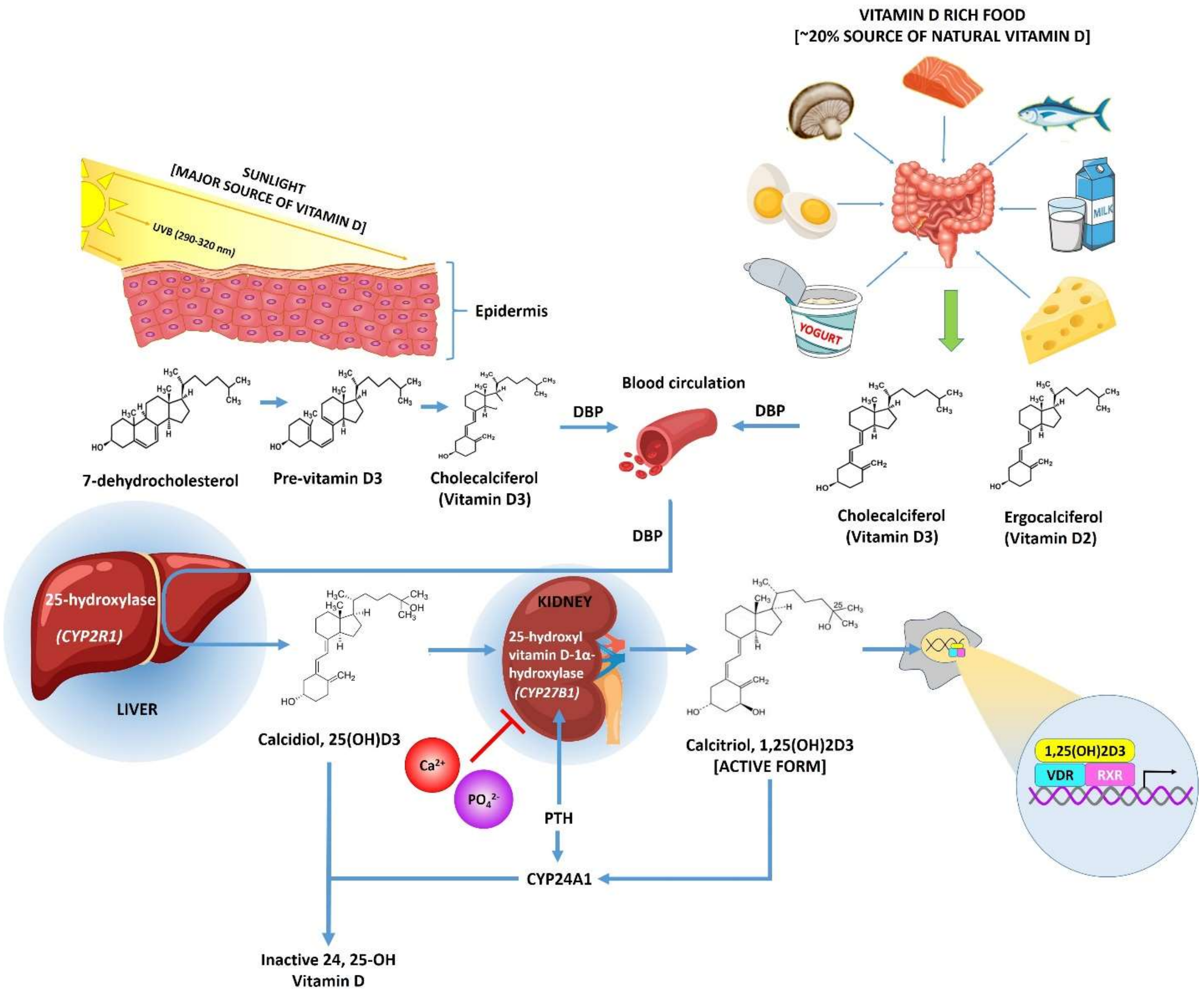

Supplementary Figure 1: Vitamin D3 (cholecalciferol) synthesis occurs in the skin, where 7-dehydrocholesterol is converted to pre-vitamin D3 in the presence of sunlight (ultraviolet B radiation; 290-320nm). Vitamin D3 and D2 (ergocalciferol) obtained from food along with vitamin D3 synthesized at skin binds to vitamin D-binding protein (DBP) in the bloodstream, and is transported to the liver where they are hydroxylated by 25-hydroxylases and converted to calcifediol or calcidiol. Calcifediol is mainly converted in the kidney to its active form 1,25$(OH)_2D$ (calcitriol) by 25-hydroxyl vitamin D-1α-hydroxylase. The calcitriol can bind to the vitamin D receptors (VDR) in the target cells to activate vitamin D response elements (VDRE) at the nuclear level. The action and synthesis of calcitriol is regulated by two key enzymes, vitamin D3 24-hydroxylase (CYP24A1) and 25-hydroxyvitamin D 1-alpha-hydroxylase (CYP27B1), which are in turn influenced by iPTH, calcium and phosphorus levels.

Supplementary Figure 2: Changes in anti-spike (S) protein antibody titers

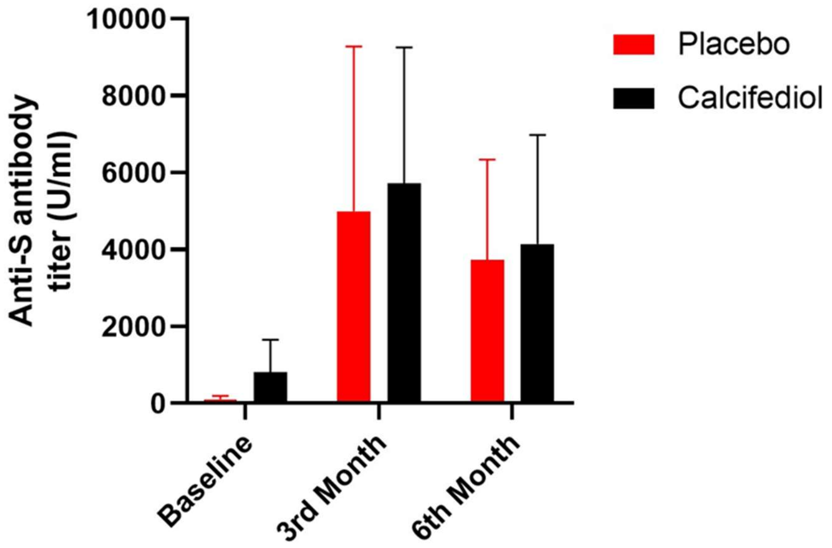

Supplementary Figure 2: Titers of anti-spike (S)-protein antibodies in placebo and calcifediol supplemented groups were measured in plasma at different time points. The data is shown as mean±standard deviation (SD).

Supplementary Figure 3: Correlation of plasma levels of vitamin D with Calcitriol

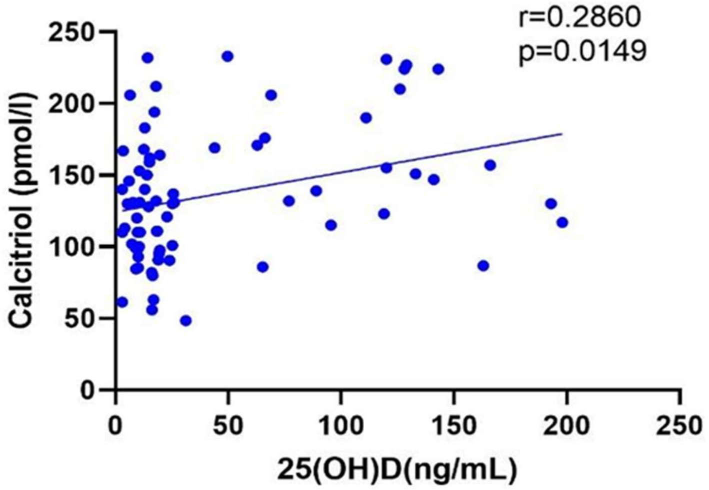

Supplementary Figure 3: Scatter plot shows a positive correlation (Spearman, r = 0.286, R^2=0.082, p= 0.0149) between 25(OH)D and Calcitriol.

Supplementary Figure 4: Combined Principal Component Analysis (PCA) of the Placebo and Calcifediol (treated) cohorts at baseline, 3rd month and 6th month.

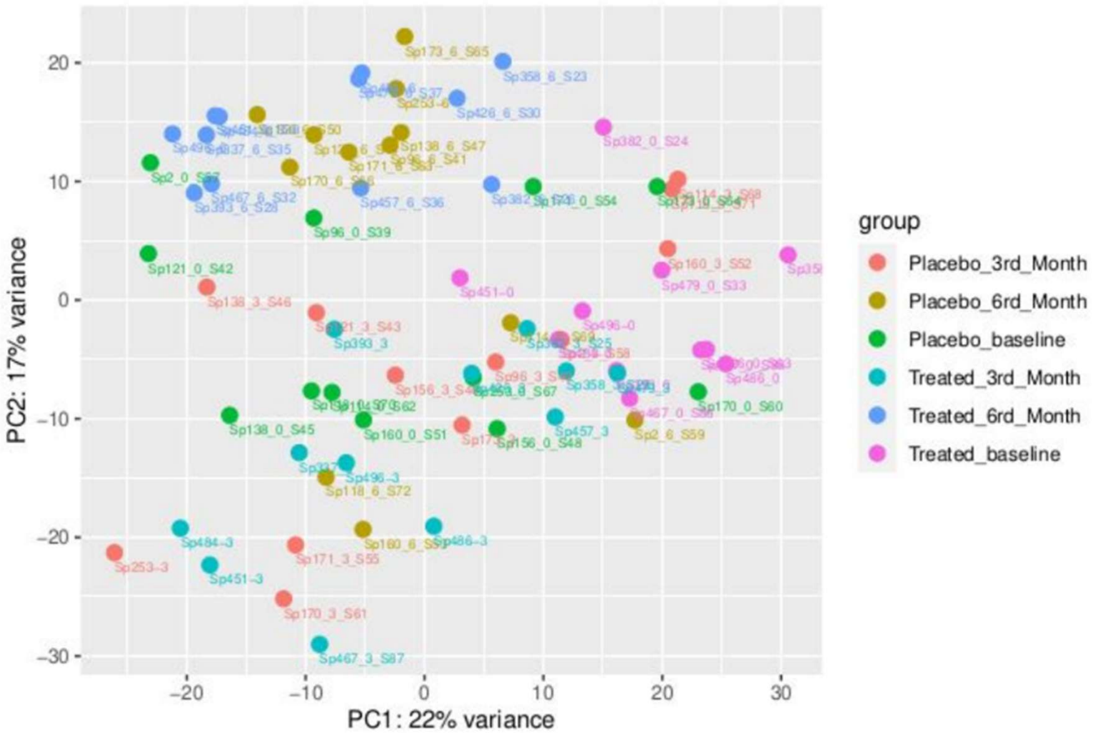

Supplementary Figure 4: Principal Component Analysis (PCA) of the treated (Calcifediol) and placebo cohort at various timepoints. Each circle represents a data point of a subject at a particular time point.

Supplementary Figure 5: Principal Component Analysis (PCA) of the Calcifediol (treated) cohort with timepoint versus age as covariant

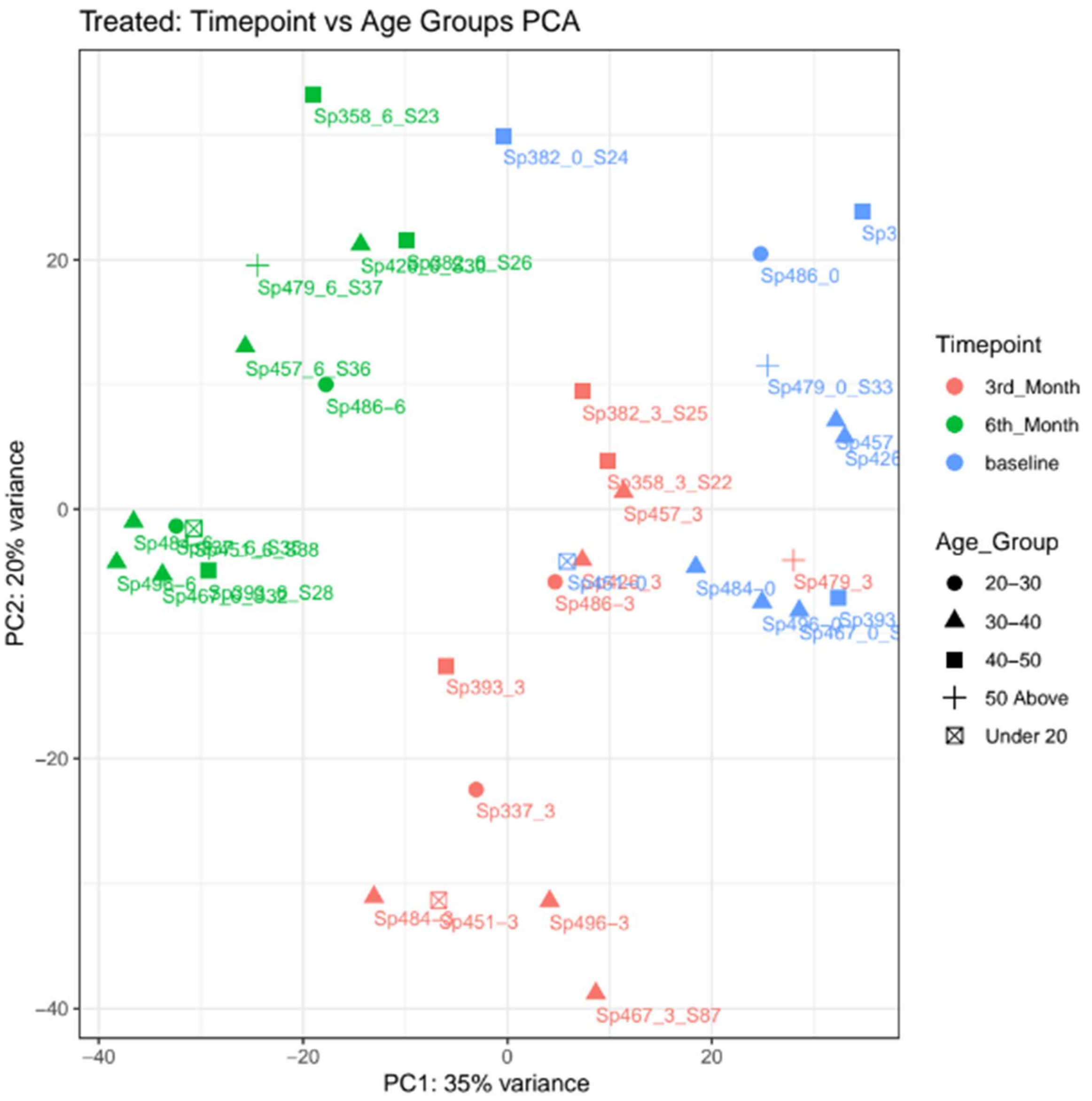

Supplementary Figure 5: Principal Component Analysis (PCA) of the Calcifediol (treated) cohort with timepoint versus age as covariant where blue-colored symbols represent baseline, red colored symbols represent 3rd month, green colored symbols represent 6th month. Circles represent individuals aged 20-30 years, triangles represent individuals aged 30-40 years, squares represent individuals aged 40-50 years, plus represents 50+ years aged individuals and cross represents individuals aged less than 20 years.

Supplementary Figure 6: Principal component analysis (PCA) of calcifediol cohort with timepoint versus BMI as covariant

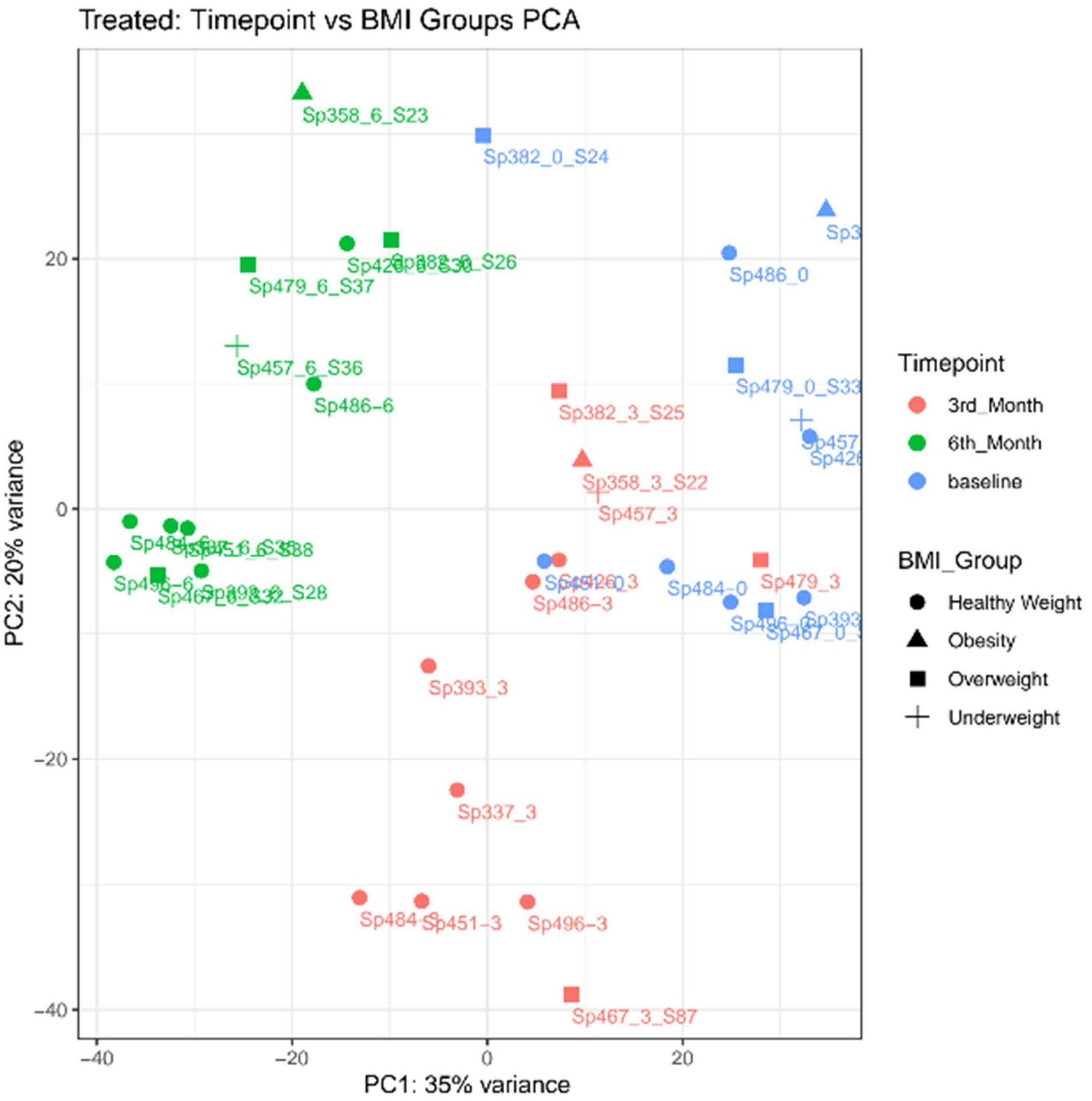

Supplementary Figure 6: Principal component analysis (PCA) of calcifediol cohort with timepoint versus BMI as covariant, where blue-colored symbols represent baseline, red colored symbols represent 3rd month, green colored symbols represent 6th month. Circles represent healthy weight individuals, triangles represent obese individuals, squares represent overweight individuals and plus represents underweight individuals.

Supplementary Figure 7: Principal Component Analysis (PCA) of calcifediol cohort with timepoint versus gender as covariant

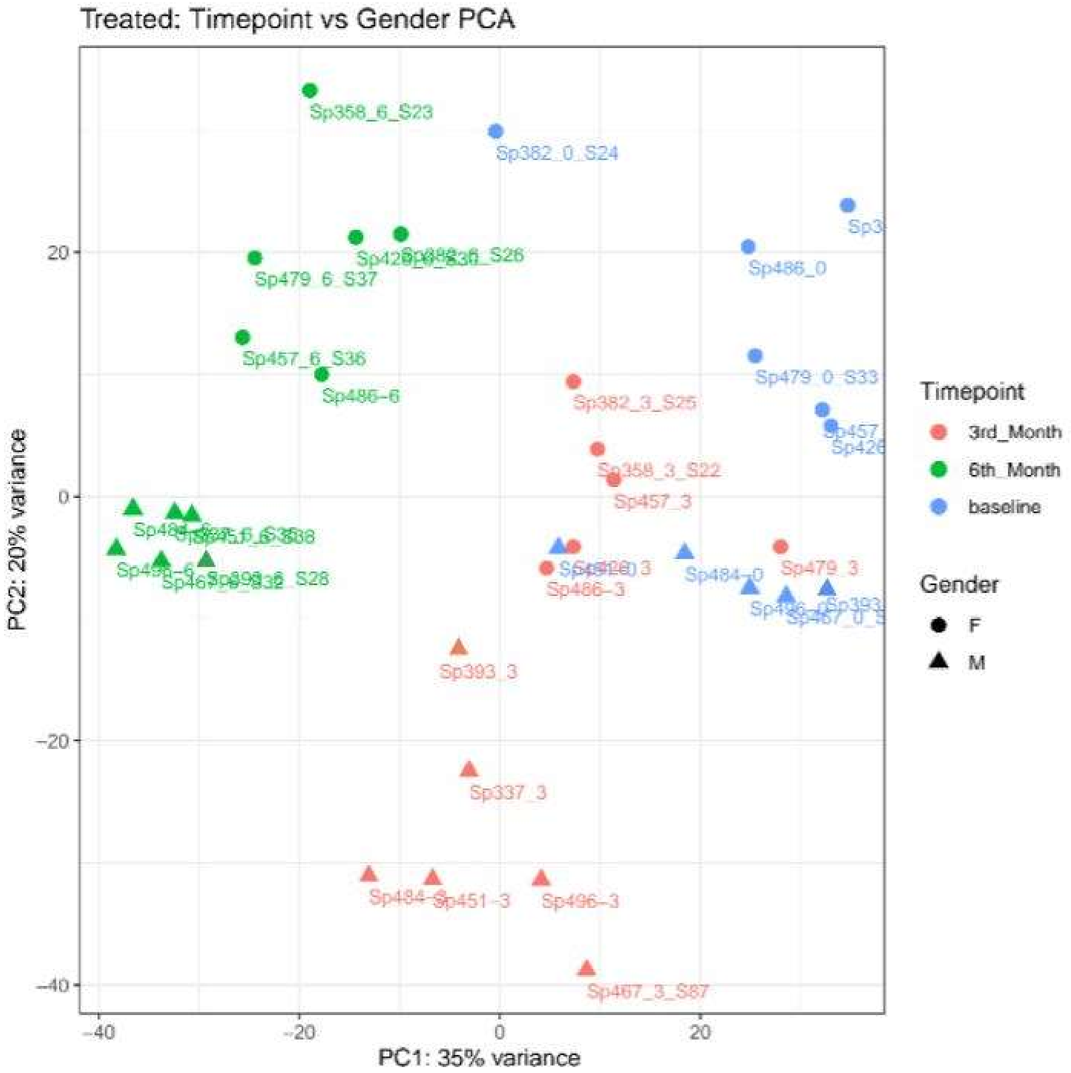

Supplementary Figure 7: Principal Component Analysis (PCA) of calcifediol cohort with timepoint versus gender as covariant where blue-colored symbols represent baseline, red colored symbols represent 3rd month, green colored symbols represent 6th month. The triangles represent males and circles represent females.

Supplementary Figure 8: Principal Component Analysis (PCA) of the Placebo cohort

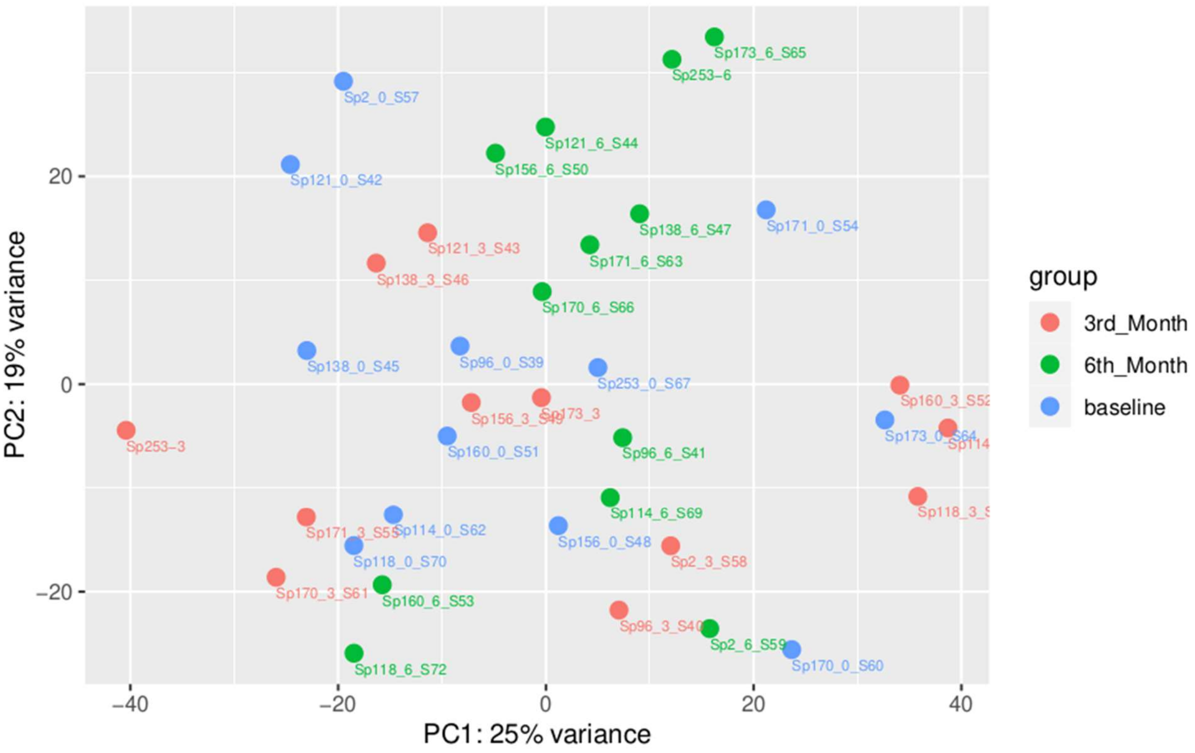

Supplementary Figure 8: Principal Component Analysis (PCA) of placebo cohort where blue bubbles represent baseline; red bubbles represent 3rd month and green bubbles represent 6th month.

Supplementary Figure 9: Time series analysis

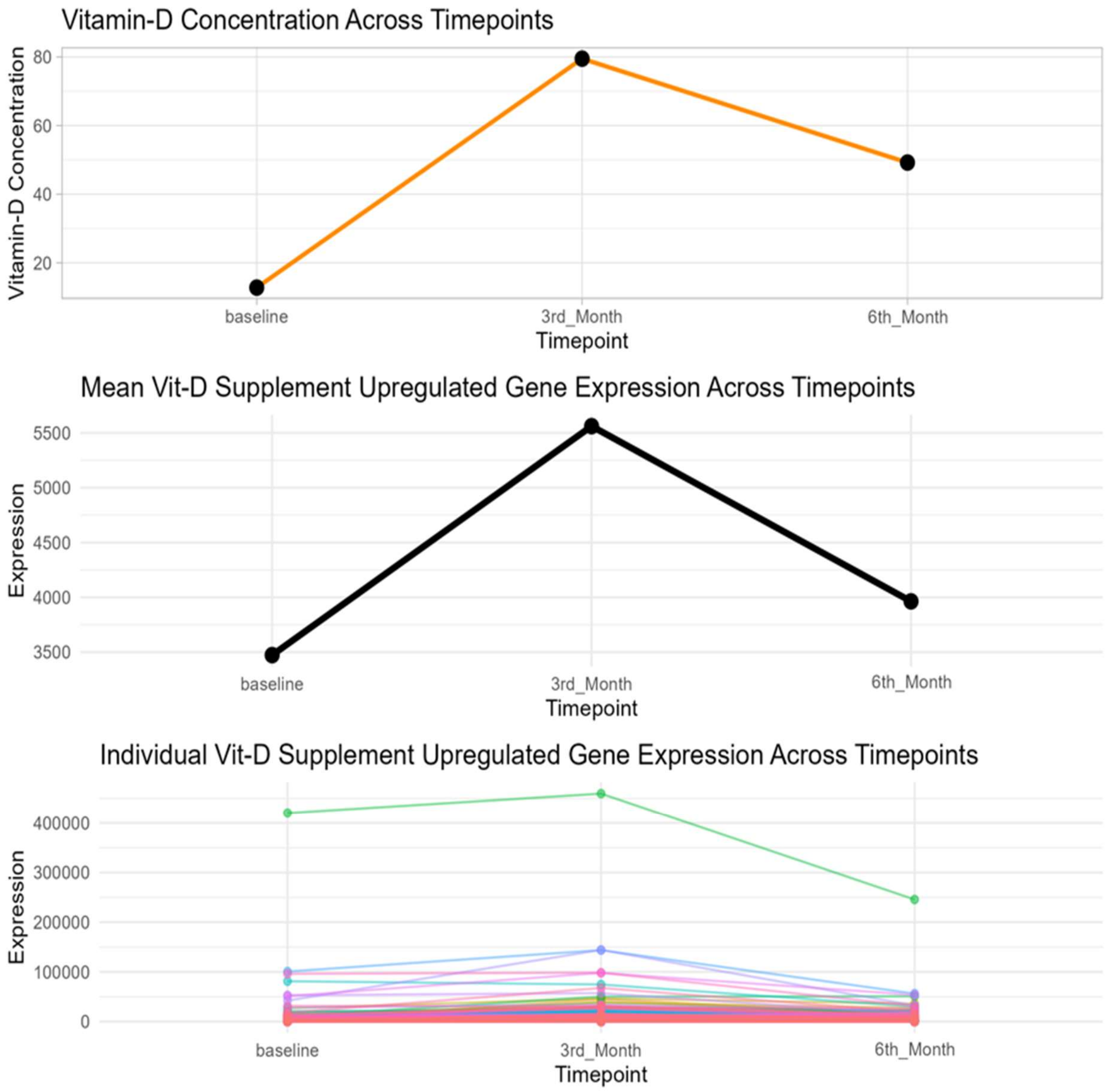

Supplementary Figure 9: Time series analysis showing the pattern of upregulation of genes (vitamin D response elements, VDREs) related to vitamin D supplementation at baseline, 3rd and 6th month in relation to vitamin D concentration in the Calcifediol supplemented (treated) group.

Supplementary Figure 10: Comparison of expression levels of *VDR, CYP27B1* and *CYP24A1*

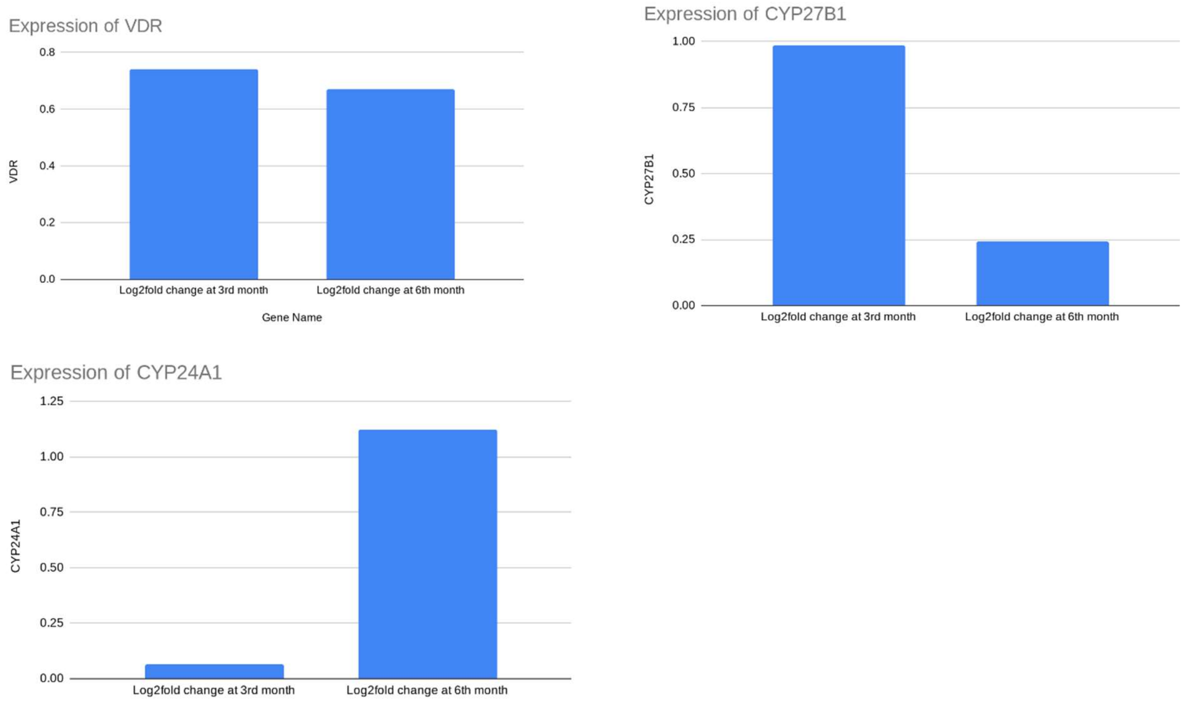

Supplementary Figure 10: Comparison of expression levels of *VDR, CYP27B1* and *CYP24A1* at 3rd month and 6th month versus baseline in the treated cohort.

Supplementary Figure 11: Downregulated pathways in calcifediol supplemented (treated) group at 6th month in comparison to baseline

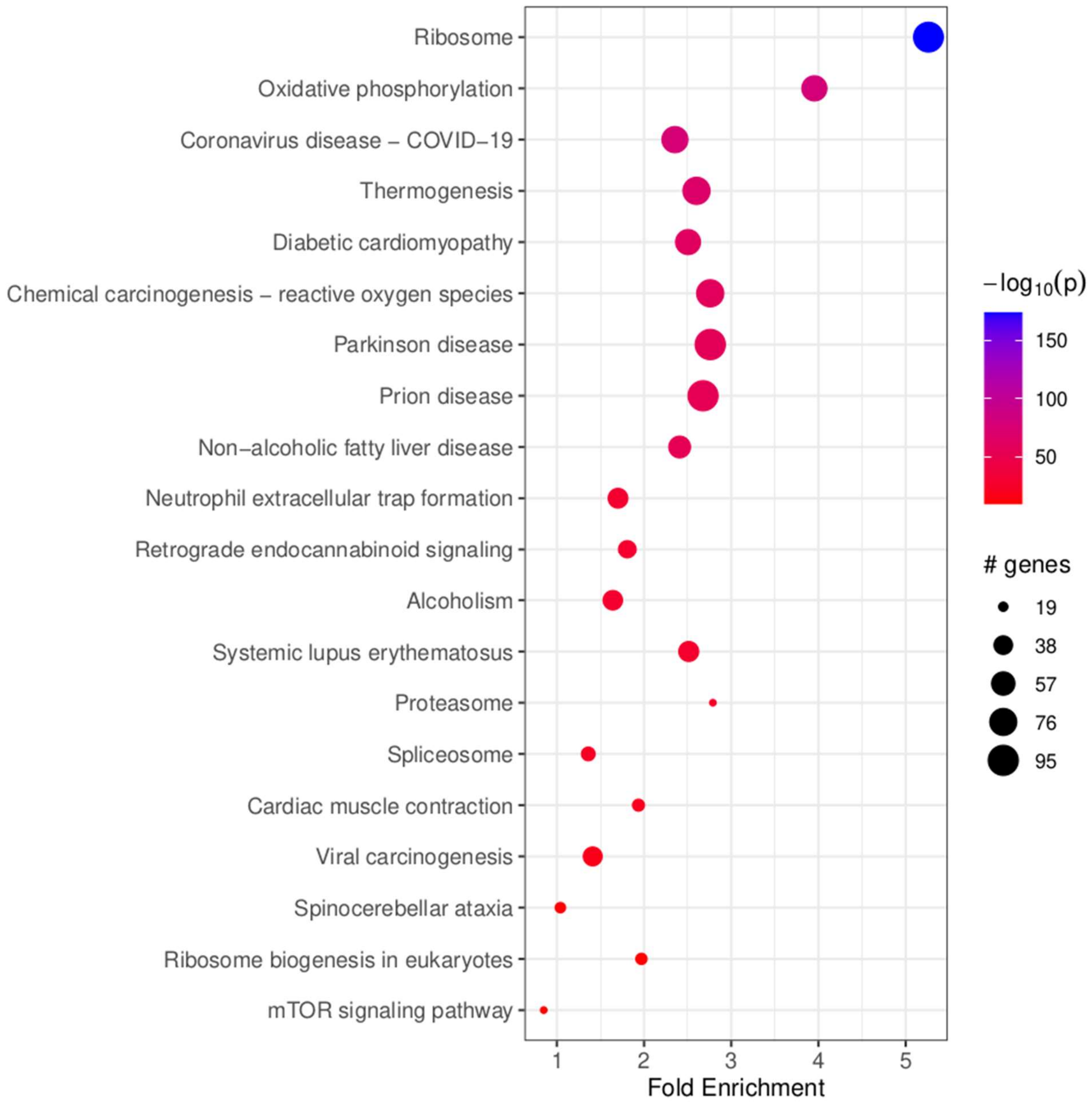

Supplementary Figure 11: Bubble plot showing KEGG analysis of downregulated pathways in calcifediol supplemented (treated) group at 6th month in comparison to baseline of the same group

Supplementary Figure 12: Downregulated pathways in calcifediol supplemented (treated) group at 6th month in comparison to 3rd month

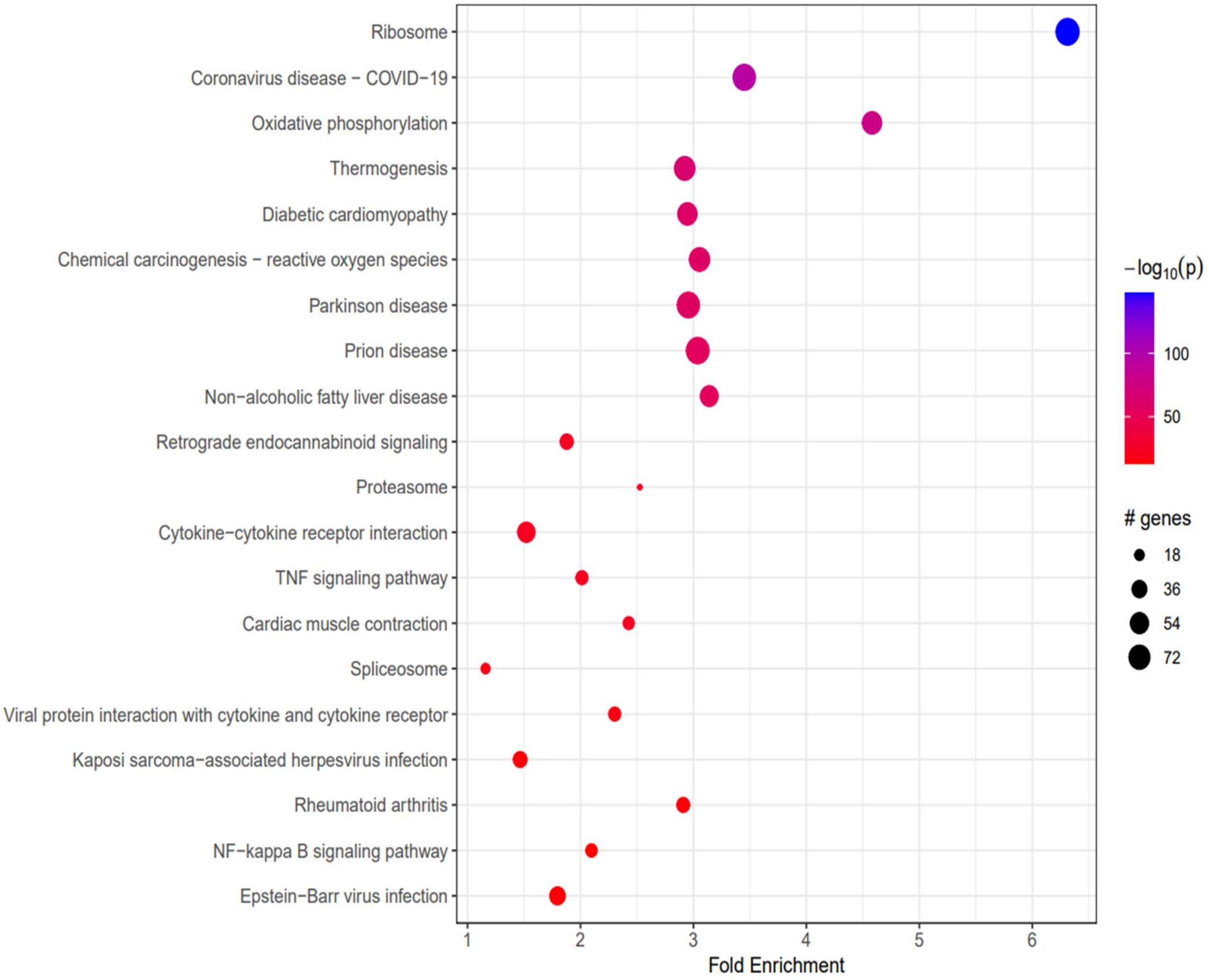

Supplementary Figure 12: Bubble plot showing KEGG analysis of downregulated pathways in calcifediol supplemented (treated) group at 6th month in comparison to 3rd month of the same group

Supplementary Figure 13: Upregulated pathways in calcifediol supplemented (treated) group at 6th month in comparison to baseline

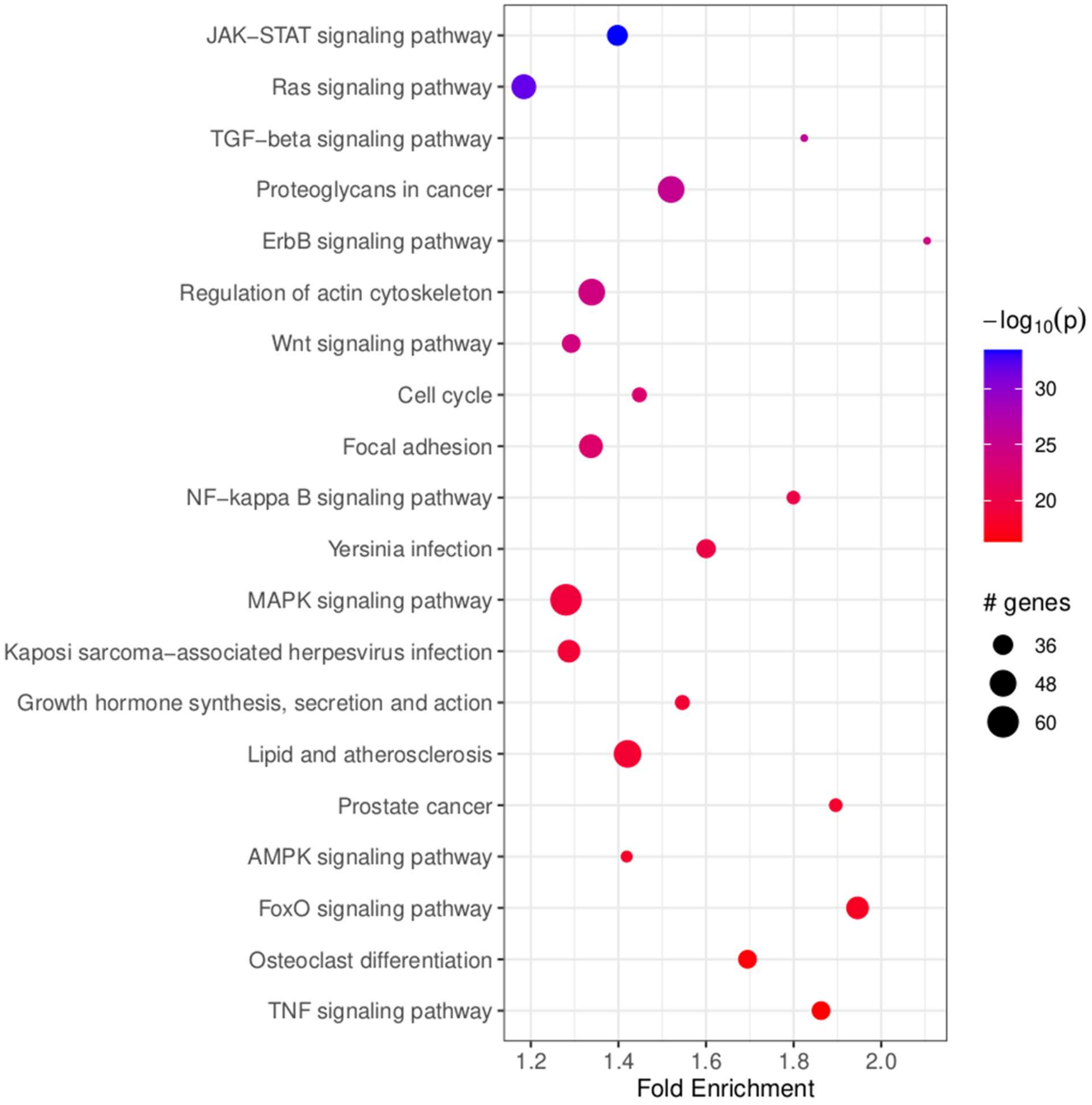

Supplementary Figure 13: Bubble plot showing KEGG analysis of upregulated pathways in calcifediol supplemented (treated) group at 6th month in comparison to baseline of the same group

Supplementary Figure 14: Upregulated pathways in calcifediol supplemented (treated) group at 6th month in comparison to 3rd month

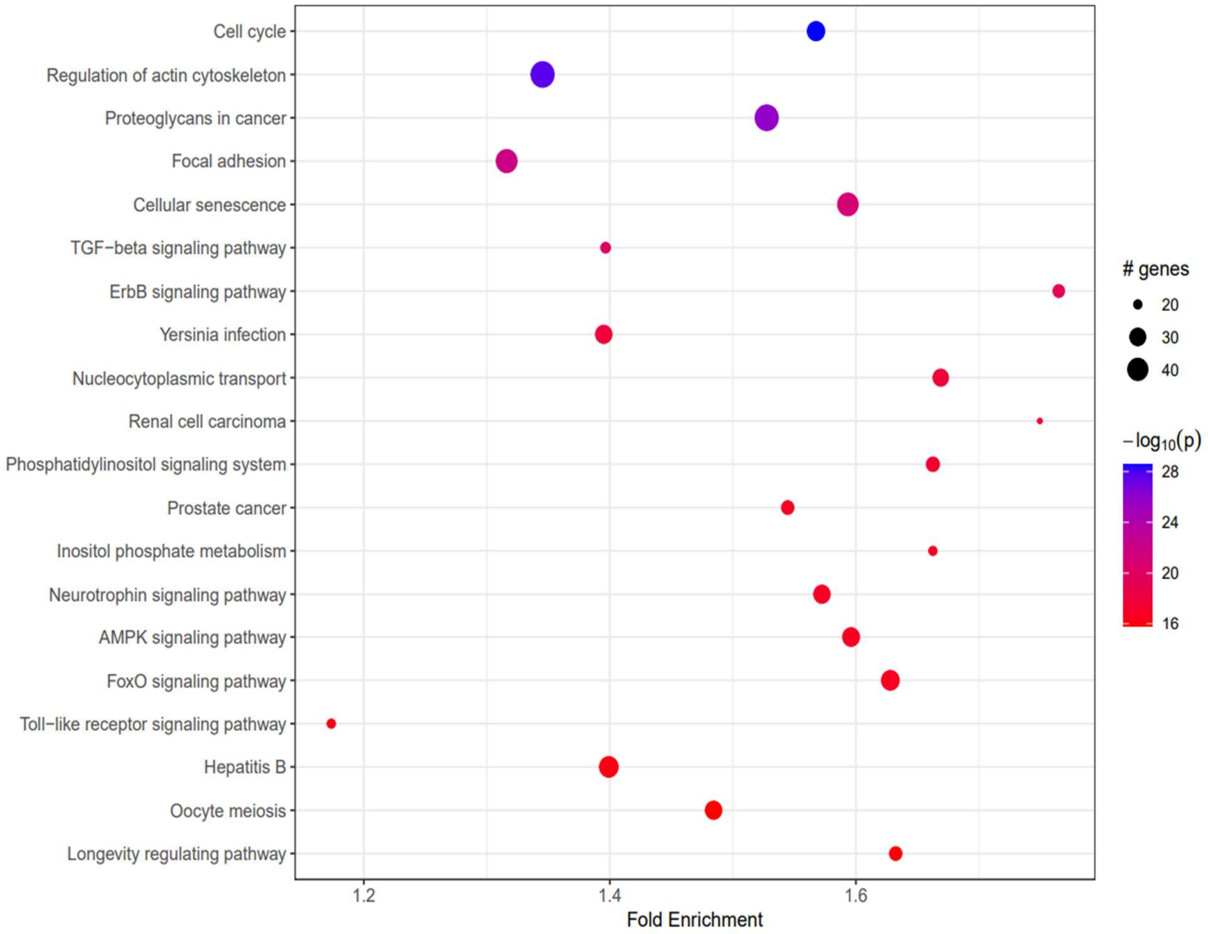

Supplementary Figure 14: Bubble plot showing KEGG analysis of upregulated pathways in calcifediol supplemented (treated) group at 6th month in comparison to 3rd month of the same group

Supplementary Table 1: The number of subjects exposed to SARS-CoV-2 and subjects with prior COVID-19 disease

Calcifediol	Placebo
**Subjects exposed to SARS-CoV-2***	
11/12	9/12
**Subjects with prior COVID-19 disease#**	
0/12	0/12

*Based on anti-N antibody positivity. #Based on patient-reported clinical symptoms consistent with COVID-19 infection

Supplementary Table 2: Calcitriol and Vitamin D levels of Calcifediol supplemented subjects

Calcifediol (N=12)			
	**Baseline**	**1st Month**	**p value**
**Plasma 25(OH)D (ng/ml)***	11.39 ± 6.9	73.20 ± 46.96	0.0006
**Plasma Calcitriol (pmol/l)#**	113.5 ± 32	176.5 ± 62.50	0.0104

The *25(OH)D and #Calcitriol levels at baseline versus 1st month were measured in plasma of calcifediol supplemented subjects. The data is shown as mean±standard deviation (SD).

Supplementary Table 3. Monotonous upregulation of vitamin D response elements (VDRE) genes at the 3rd and 6th month in the calcifediol supplemented (treated) group.

Gene ID_Gene Name
ENSG00000090104_*RGS1*
ENSG00000163874_*ZC3H12A*
ENSG00000143420_*ENSA*
ENSG00000162910_*MRPL55*
ENSG00000116898_*MRPS15*
ENSG00000158615_*PPP1R15B*
ENSG00000116560_*SFPQ*
ENSG00000162711_*NLRP3*
ENSG00000143514_*TP53BP2*
ENSG00000171865_*RNASEH1*
ENSG00000136718_*IMP4*

ENSG00000125633_*CCDC93*
ENSG00000264229_*RNU4ATAC*
ENSG00000136541_*ERMN*
ENSG00000115520_*COQ10B*
ENSG00000168883_*USP39*
ENSG00000136689_*IL1RN*
ENSG00000075426_*FOSL2*
ENSG00000162924_*REL*
ENSG00000176142_*TMEM39A*
ENSG00000163584_*RPL22L1*
ENSG00000163659_*TIPARP*
ENSG00000134107_*BHLHE40*
ENSG00000144597_*EAF1*
ENSG00000144741_*SLC25A26*
ENSG00000114023_*FAM162A*
ENSG00000136603_*SKIL*
ENSG00000145425_*RPS3A*
ENSG00000081041_*CXCL2*
ENSG00000138670_*RASGEF1B*
ENSG00000152409_*JMY*
ENSG00000164611_*PTTG1*
ENSG00000082515_*MRPL22*
ENSG00000161011_*SQSTM1*
ENSG00000198055_*GRK6*
ENSG00000113356_*POLR3G*
ENSG00000171617_*ENC1*
ENSG00000145632_*PLK2*
ENSG00000224032_*EPB41L4A-AS1*
ENSG00000113070_*HBEGF*
ENSG00000078401_*EDN1*
ENSG00000145979_*TBC1D7*
ENSG00000120437_*ACAT2*
ENSG00000221821_*C6orf226*
ENSG00000156508_*EEF1A1*
ENSG00000130340_*SNX9*
ENSG00000111832_*RWDD1*
ENSG00000118503_*TNFAIP3*
ENSG00000169976_*SF3B5*
ENSG00000124562_*SNRPC*
ENSG00000232810_*TNF*
ENSG00000112715_*VEGFA*

ENSG00000186480_*INSIG1*
ENSG00000135245_*HILPDA*
ENSG00000105821_*DNAJC2*
ENSG00000128524_*ATP6V1F*
ENSG00000106245_*BUD31*
ENSG00000134594_*RAB33A*
ENSG00000147224_*PRPS1*
ENSG00000164758_*MED30*
ENSG00000070501_*POLB*
ENSG00000137074_*APTX*
ENSG00000107175_*CREB3*
ENSG00000148303_*RPL7A*
ENSG00000123975_*CKS2*
ENSG00000085117_*CD82*
ENSG00000174744_*BRMS1*
ENSG00000162236_*STX5*
ENSG00000149806_*FAU*
ENSG00000120539_*MASTL*
ENSG00000265354_*TIMM23*
ENSG00000108179_*PPIF*
ENSG00000165997_*ARL5B*
ENSG00000129315_*CCNT1*
ENSG00000133773_*CCDC59*
ENSG00000167550_*RHEBL1*
ENSG00000136003_*ISCU*
ENSG00000175197_*DDIT3*
ENSG00000150991_*UBC*
ENSG00000110848_*CD69*
ENSG00000089818_*NECAP1*
ENSG00000211791_*TRAV13-2*
ENSG00000166920_*C15orf48*
ENSG00000185043_*CIB1*
ENSG00000134419_*RPS15A*
ENSG00000103257_*SLC7A5*
ENSG00000102984_*ZNF821*
ENSG00000140743_*CDR2*
ENSG00000103035_*PSMD7*
ENSG00000109113_*RAB34*
ENSG00000101544_*ADNP2*
ENSG00000124216_*SNAI1*
ENSG00000101413_*RPRD1B*

ENSG00000125812_*GZF1*
ENSG00000105058_*FAM32A*
ENSG00000011422_*PLAUR*
ENSG00000169021_*UQCRFS1*
ENSG00000167671_*UBXN6*
ENSG00000130332_*LSM7*
ENSG00000076924_*XAB2*
ENSG00000142541_*RPL13A*
ENSG00000173875_*ZNF791*
ENSG00000130844_*ZNF331*
ENSG00000160570_*DEDD2*
ENSG00000130255_*RPL36*
ENSG00000186431_*FCAR*
ENSG00000099985_*OSM*
ENSG00000198355_*PIM3*
ENSG00000100362_*PVALB*
ENSG00000269220_*LINC00528*
ENSG00000100410_*PHF5A*
ENSG00000128228_*SDF2L1*
ENSG00000185022_*MAFF*
ENSG00000157557_*ETS2*

Supplementary Table 4: Complete List of Biological processes found differentially upregulated upon Calcifediol supplementation (Treated 6 months versus treated baseline).

**Biological Process**
Protein deubiquitination
Protein ubiquitination
Cellular response to DNA damage stimulus
Protein K63-linked deubiquitination
Centriole replication
Regulation of transcription by RNA polymerase II
Chromatin remodeling
Protein K48-linked deubiquitination
Hippo signaling
Regulation of small GTPase mediated signal transduction

Ubiquitin-dependent protein catabolic process
Peptidyl-serine phosphorylation
RNA splicing
COPII-coated vesicle cargo loading
Regulation of DNA-templated transcription
Protein autophosphorylation
Regulation of cell cycle
Double-strand break repair
Alternative mRNA splicing, via spliceosome
miRNA processing
miRNA-mediated gene silencing by inhibition of translation
Positive regulation of I-kappa B kinase/NF-kappa B signaling
Clathrin coat assembly
Protein localization to centrosome
Protein polyubiquitination
Proteasome-mediated ubiquitin-dependent protein catabolic process
mRNA export from nucleus
Negative regulation of translation
Regulation of alternative mRNA splicing, via spliceosome
Mitotic spindle assembly
Positive regulation of NF-kappa B transcription factor activity
BMP signaling pathway
Intrinsic apoptotic signaling pathway
Double-strand break repair via homologous recombination
DNA repair
Regulation of signal transduction by p53 class mediator
Intracellular signal transduction
RNA processing
Microtubule cytoskeleton organization

Regulation of circadian rhythm
Post-transcriptional regulation of gene expression
Ephrin receptor signaling pathway
Nuclear pore complex assembly
SCF-dependent proteasomal ubiquitin-dependent protein catabolic process
Histone H4 acetylation
Cellular response to insulin stimulus
mRNA splice site selection
mRNA processing
Circadian regulation of gene expression
Regulation of mRNA stability
Protein K11-linked deubiquitination
Peptidyl-threonine phosphorylation
Positive regulation of DNA-templated transcription, elongation
Histone H3 acetylation
Positive regulation of DNA repair
Epidermal growth factor receptor signaling pathway
Negative regulation of cilium assembly
Cell migration
Memory T cell responses
Positive regulation of translation
Centrosome duplication
Endoplasmic reticulum to Golgi vesicle-mediated transport
Positive regulation of double-strand break repair via homologous recombination
Golgi organization
Positive regulation of transcription by RNA polymerase III
Substrate adhesion-dependent cell spreading
Response to ionizing radiation
Phosphatidylinositol phosphate biosynthetic process
Histone H2A acetylation

Histone deacetylation
DNA duplex unwinding
Mitotic cytokinesis
Stress granule assembly
Positive regulation of protein ubiquitination
Protein acetylation
Activation of GTPase activity
G1/S transition of mitotic cell cycle
Positive regulation of nuclear-transcribed mRNA catabolic process, deadenylation-dependent decay
Cytokine-mediated signaling pathway
mRNA transcription by RNA polymerase II
Protein stabilization
DNA replication
mRNA splicing, via spliceosome
Transcription initiation-coupled chromatin remodeling
RNA polymerase II preinitiation complex assembly
Regulation of mRNA splicing, via spliceosome
Retrograde transport, endosome to Golgi
Mismatch repair
Negative regulation of NF-kappa B transcription factor activity
Phosphorylation
Cellular response to ionizing radiation
Insulin receptor signaling pathway
mRNA destabilization
Protein K48-linked ubiquitination
Protein K11-linked ubiquitination
Protein localization to plasma membrane
Intracellular estrogen receptor signaling pathway
Nucleotide-excision repair
Positive regulation of transcription initiation by RNA polymerase II

Positive regulation of cytokinesis
Actin cytoskeleton organization
Regulation of cellular response to heat
Histone H3 deacetylation
Positive regulation of blood vessel endothelial cell migration
Positive regulation of phosphatidylinositol 3-kinase signaling
Positive regulation of nuclear-transcribed mRNA poly(A) tail shortening
Protein sumoylation
Positive regulation of extrinsic apoptotic signaling pathway
Replication fork processing
Mitotic G2 DNA damage checkpoint signaling
Positive regulation of telomere maintenance via telomerase
Cellular response to UV
Positive regulation of canonical Wnt signaling pathway
Protein autoubiquitination
Localization
Pre-miRNA processing
Regulation of ventricular cardiac muscle cell action potential
Positive regulation of microtubule polymerization
Amyloid fibril formation
Regulation of DNA replication
Positive regulation of GTPase activity
Positive regulation of interferon-alpha production
Androgen receptor signaling pathway
Negative regulation of gene expression
Synaptic vesicle endocytosis
Microvillus assembly
Chromatin organization
Positive regulation of glucose import

DNA replication initiation
Insulin-like growth factor receptor signaling pathway
Positive regulation of DNA-binding transcription factor activity
Regulation of heart rate by cardiac conduction
Mitotic spindle organization
Intrinsic apoptotic signaling pathway in response to DNA damage by p53 class mediator
Phosphatidylinositol 3-kinase signaling
Cell adhesion
Double-strand break repair via nonhomologous end joining
Integrin-mediated signaling pathway
Protein import into nucleus
Positive regulation of mRNA catabolic process
Negative regulation of proteasomal ubiquitin-dependent protein catabolic process
Positive regulation of miRNA-mediated gene silencing
Mitotic spindle assembly checkpoint signaling
Negative regulation of anoikis
Negative regulation of stress fiber assembly
Negative regulation of viral genome replication
Positive regulation of isotype switching
Protein K63-linked ubiquitination
Protein destabilization
Positive regulation of lipid biosynthetic process
Positive regulation of lamellipodium assembly
Positive regulation of cytoplasmic translation
Endoplasmic reticulum organization
Autophagosome maturation
Negative regulation of I-kappa B kinase/NF-kappa B signaling
Interleukin-1-mediated signaling pathway
Regulation of hematopoietic stem cell differentiation
Telomere maintenance

Mitotic cell cycle
Positive regulation of transcription elongation by RNA polymerase II
Cilium assembly
Negative regulation of microtubule depolymerization
Protein dephosphorylation
Response to UV
Clathrin-dependent endocytosis
MAPK cascade
Positive regulation of miRNA transcription
Stimulatory C-type lectin receptor signaling pathway
Response to lipopolysaccharide
Negative regulation of protein ubiquitination
Cellular response to amino acid starvation
Response to estrogen
Regulation of embryonic development
Cellular glucose homeostasis
Ubiquitin-dependent ERAD pathway
Regulation of DNA strand elongation
Positive regulation of protein localization to plasma membrane
Nuclear-transcribed mRNA catabolic process, nonsense-mediated decay
Golgi to endosome transport
Cellular response to starvation
Notch signaling pathway
3'-UTR-mediated mRNA destabilization
Transforming growth factor beta receptor signaling pathway
Positive regulation of histone H3-K4 methylation
B cell receptor signaling pathway
Regulation of mitotic cell cycle
Cholesterol biosynthetic process
Cellular response to gamma radiation

Positive regulation of apoptotic process
Positive regulation of SMAD protein signal transduction
Cell adhesion mediated by integrin
Ion transmembrane transport
Transcription initiation at RNA polymerase II promoter
Positive regulation of epithelial cell migration
Regulation of chromosome organization
Positive regulation of epithelial to mesenchymal transition
Apoptotic process
Cellular senescence
Mitochondrion organization
Negative regulation of cell growth
Intracellular protein transport
Protein-containing complex assembly
Spliceosomal complex assembly
Positive regulation of interferon-beta production
Vascular endothelial growth factor receptor signaling pathway
Positive regulation of angiogenesis
Response to interleukin-1
Positive regulation of protein kinase activity
Maintenance of protein location in nucleus
Post-Golgi vesicle-mediated transport
Regulation of RNA splicing
Negative regulation of canonical Wnt signaling pathway
Peptidyl-tyrosine phosphorylation
Cellular response to hypoxia
Regulation of tubulin deacetylation
Cytoskeleton organization
Sister chromatid cohesion
Positive regulation of mitotic cell cycle
Monoubiquitinated histone H2A deubiquitination

Positive regulation of RNA splicing
Ras protein signal transduction
Positive regulation of double-strand break repair via nonhomologous end joining
SMAD protein signal transduction
Cellular response to tumor necrosis factor
Monoubiquitinated histone deubiquitination
Protein localization to chromatin
Positive regulation of peptidyl-serine phosphorylation
Circadian rhythm
Histone modification
Peptidyl-tyrosine dephosphorylation
Positive regulation of protein phosphorylation
Positive regulation of rRNA processing
Regulation of tumor necrosis factor-mediated signaling pathway
Epithelial to mesenchymal transition
Positive regulation of RIG-I signaling pathway
Heterochromatin formation
Lymphangiogenesis
Centriole-centriole cohesion
Vesicle-mediated transport
Response to virus
Positive regulation of bone mineralization
Regulation of endocytosis
Negative regulation of ubiquitin-protein transferase activity
Positive regulation of protein binding
Lysosome organization
Regulation of glycolytic process
Regulation of androgen receptor signaling pathway
Regulation of epithelial to mesenchymal transition
Positive regulation of filopodium assembly

Cellular response to glucose starvation
Macro-autophagy
Microtubule nucleation
Establishment of protein localization
Protein auto-ADP-ribosylation
Mitochondrion morphogenesis
Calcium ion transmembrane transport
Mitotic metaphase plate congression
Regulation of dendrite morphogenesis
Negative regulation of double-strand break repair via homologous recombination
Activation of innate immune response
Positive regulation of T cell chemotaxis
Nucleosome disassembly
Transcription by RNA polymerase II
Cellular response to glucocorticoid stimulus
DNA recombination
Defense response to virus
Negative regulation of translational initiation
Regulation of innate immune response
Bile acid biosynthetic process
Chloride transmembrane transport
Ribosome biogenesis
Mitochondrial fission
Regulation of microtubule cytoskeleton organization
Exonucleolytic catabolism of deadenylated mRNA
Positive regulation of T cell cytokine production
Negative regulation of cell adhesion
Negative regulation of mRNA splicing, via spliceosome
Regulation of translation
Nucleocytoplasmic transport
Negative regulation of circadian rhythm

Mirna-mediated gene silencing
Cellular response to exogenous dsRNA
Base-excision repair
Response to endoplasmic reticulum stress
Negative regulation of intracellular estrogen receptor signaling pathway
Positive regulation of NLRP3 inflammasome complex assembly
Regulation of chromatin organization
Ventricular septum morphogenesis
Receptor internalization
Positive regulation of G1/S transition of mitotic cell cycle
Regulation of focal adhesion assembly
Positive regulation of transforming growth factor beta receptor signaling pathway
Establishment of endothelial intestinal barrier
Negative regulation of type I interferon production
Regulation of mitotic spindle assembly
Regulation of autophagy of mitochondrion
Primary miRNA processing
Fat cell differentiation
Cellular response to chemokine
Positive regulation of erythrocyte differentiation
Positive regulation of viral genome replication
Positive regulation of pathway-restricted SMAD protein phosphorylation
CRD-mediated mRNA stabilization
Regulation of Golgi organization
Positive regulation of Wnt signaling pathway
Cellular response to virus
Positive regulation of NIK/NF-kappa B signaling
Regulation of autophagy
Positive regulation of JNK cascade
Regulation of chromosome segregation

Membrane protein ectodomain proteolysis
Negative regulation of DNA replication
Trans lesion synthesis
Negative regulation of autophagy
Protein deacetylation
Positive regulation of cell migration
Phosphatidylinositol dephosphorylation
Nuclear-transcribed mRNA catabolic process
Myd88-dependent toll-like receptor signaling pathway
Regulation of cytokinesis
Long-chain fatty-acyl-CoA biosynthetic process
Early endosome to late endosome transport
Negative regulation of protein kinase activity
Heart development
Cellular response to estradiol stimulus
Negative regulation of DNA-binding transcription factor activity
Response to hypoxia
Negative regulation of hippo signaling
Cell-substrate adhesion
Positive regulation by host of viral transcription
Microtubule depolymerization
Protein monoubiquitination
Erythrocyte differentiation
DNA damage response, signal transduction by p53 class mediator
Positive regulation of osteoblast differentiation
Interleukin-6-mediated signaling pathway
Membrane organization
Activation of NF-kappa B-inducing kinase activity
Replicative senescence
ERAD pathway
Protein O-linked glycosylation

Exocytosis
DNA damage checkpoint signaling
Histone H4-K16 acetylation
T cell receptor signaling pathway
Establishment or maintenance of cell polarity
Regulation of cell population proliferation
Regulation of cell growth
Positive regulation of insulin receptor signaling pathway
Peroxisome organization
Regulation of stress fiber assembly
Regulation of mitotic spindle organization
Negative regulation of cysteine-type endopeptidase activity involved in apoptotic process
Negative regulation of DNA damage response, signal transduction by p53 class mediator
Spindle organization
Positive regulation of autophagy
Cell-matrix adhesion
Positive regulation of transcription by RNA polymerase I
Positive regulation of cysteine-type endopeptidase activity involved in apoptotic process
Stress-activated MAPK cascade
Establishment of epithelial cell apical/basal polarity
Positive regulation of protein localization to nucleus
DNA synthesis involved in DNA repair
Positive regulation of substrate adhesion-dependent cell spreading
Negative regulation of cell cycle
Protein modification process
Cellular response to cholesterol
Signal transduction in response to DNA damage
Ion transport
Peptidyl-threonine dephosphorylation
Regulation of filopodium assembly

Negative regulation of protein kinase B signaling
Response to insulin
Endoplasmic reticulum calcium ion homeostasis
miRNA-mediated gene silencing by mRNA destabilization
Axon extension

Supplementary Table 5: Complete list of biological functions found differentially downregulated upon Calcifediol supplementation (Treated 6 months versus treated baseline)

**Biological process**
Mitochondrial respiratory chain complex I assembly
Cytoplasmic translation
rRNA processing
Translation
mRNA splicing, via spliceosome
Mitochondrial translation
Ribosomal small subunit biogenesis
Transcription by RNA polymerase II
Tumor necrosis factor-mediated signaling pathway
Protein N-linked glycosylation
Ribosomal large subunit biogenesis
Aerobic respiration
Mitochondrion organization
Interleukin-1-mediated signaling pathway
Translational initiation
Protein stabilization
Protein targeting to mitochondrion
Tail-anchored membrane protein insertion into ER membrane

Nucleotide-excision repair
Protein insertion into ER membrane by stop-transfer membrane-anchor sequence
Positive regulation of NF-kappa B transcription factor activity
Generation of precursor metabolites and energy
SCF-dependent proteasomal ubiquitin-dependent protein catabolic process
Antigen processing and presentation of exogenous peptide antigen via MHC class II
Positive regulation of DNA-binding transcription factor activity
Endoplasmic reticulum to Golgi vesicle-mediated transport
Positive regulation of protein phosphorylation
Negative regulation of protein ubiquitination
Positive regulation of NIK/NF-kappa B signaling
Positive regulation of actin cytoskeleton reorganization
Negative regulation of cell growth
Positive regulation of mitochondrial fission
Positive regulation of I-kappa B kinase/NF-kappa B signaling
Release of cytochrome c from mitochondria
Positive regulation of translation
Extrinsic apoptotic signaling pathway via death domain receptors
RNA splicing
Regulation of apoptotic process
Regulation of mitochondrion organization
Protein modification process
Protein peptidyl-prolyl isomerization
Positive regulation of protein-containing complex assembly
Cell division

Negative regulation of protein binding
Proteolysis
Antimicrobial humoral immune response mediated by antimicrobial peptide
Spliceosomal tri-snRNP complex assembly
Positive regulation of inflammatory response
Response to oxidative stress
Negative regulation of NF-kappa B transcription factor activity
Positive regulation of ruffle assembly
Defense response to Gram-positive bacterium
Endocytic recycling
Mitochondrial electron transport, NADH to ubiquinone
Negative regulation of transcription elongation by RNA polymerase II
Negative regulation of amyloid fibril formation
Defense response to fungus
TOR signaling
Protein insertion into mitochondrial inner membrane
Negative regulation of protein kinase activity
Positive regulation of intrinsic apoptotic signaling pathway
Regulation of autophagy of mitochondrion
Negative regulation of ubiquitin-dependent protein catabolic process
Glycolytic process
Positive regulation of leukocyte adhesion to vascular endothelial cell
Ubiquitin-dependent ERAD pathway
Protein folding
Response to virus
Mitochondrial fission
Mitochondrial fusion

Protein ubiquitination
Phosphorylation
Positive regulation of heterotypic cell-cell adhesion
Ras protein signal transduction
Positive regulation of T cell mediated cytotoxicity
DNA repair
Protein refolding
Regulation of signal transduction by p53 class mediator
Intracellular signal transduction
Negative regulation of proteolysis
Cellular response to oxidative stress
Negative regulation of viral transcription
Lysosome localization
Negative regulation of phosphorylation
Lipopolysaccharide-mediated signaling pathway
Negative regulation of viral entry into host cell
Killing of cells of another organism
Cellular response to nerve growth factor stimulus
Neutrophil chemotaxis
Doxorubicin metabolic process
Regulation of mitochondrial membrane potential
Chaperone cofactor-dependent protein refolding
Regulation of protein stability
Chromatin remodeling
Glutathione metabolic process
Peptidyl-tyrosine phosphorylation
Protein destabilization

Negative regulation of endopeptidase activity
Protein K63-linked ubiquitination
Response to type II interferon
Mitochondrial cytochrome c oxidase assembly
Supramolecular fiber organization
Macroautophagy
Cellular senescence
Regulation of G1/S transition of mitotic cell cycle
Positive regulation of protein localization to plasma membrane
Positive regulation by host of viral process
Neuron apoptotic process
I-kappaB kinase/NF-kappaB signaling
Cell surface receptor signaling pathway
Peptidyl-serine dephosphorylation
Endosome organization
Mitophagy
Spliceosomal snrnp assembly
Positive regulation of p38mapk cascade
Regulation of cell cycle
Regulation of DNA strand elongation
Positive regulation of peptidyl-serine phosphorylation
Protein localization to plasma membrane
Antiviral innate immune response
Positive regulation of cholesterol efflux
Positive regulation of protein metabolic process
Protein sumoylation
Regulation of I-kappa B kinase/NF-kappa B signaling

Negative regulation of release of cytochrome c from mitochondria
Negative regulation of receptor signaling pathway via JAK-STAT
T cell chemotaxis
Cellular calcium ion homeostasis
Endosome to lysosome transport
Regulation of chromosome organization
Regulation of focal adhesion assembly
DNA damage response, signal transduction by p53 class mediator resulting in cell cycle arrest
Regulation of macroautophagy
Cellular response to tumor necrosis factor
Positive regulation of protein localization to nucleus
Response to interferon-beta
Cellular response to lipopolysaccharide
Positive regulation of fibroblast proliferation
Cellular response to heat
Histone H2A acetylation
Establishment of skin barrier
Positive regulation of DNA repair
Regulation of circadian rhythm
MAPK cascade
Negative regulation of extrinsic apoptotic signaling pathway
Transmembrane receptor protein tyrosine kinase signaling pathway
T cell costimulation
Positive regulation of smooth muscle cell proliferation
Response to interferon-alpha
Retrograde vesicle-mediated transport, Golgi to endoplasmic reticulum

Regulation of mitochondrial membrane permeability
Regulation of glycolytic process
Defense response to Gram-negative bacterium
Actin cytoskeleton organization
Activation of cysteine-type endopeptidase activity involved in apoptotic process
Antibacterial humoral response
Positive regulation of T cell activation

Supplementary Table 6: Assay ID and reporter dyes of the Taqman primers and probes used in the Gene Expression Analysis

GENE	ASSAY ID	REPORTER DYE
BCL6	Hs00153368_m1	FAM
EOMES	Hs00172872_m1	
ID3	Hs00171409_m1	
IL7Rα	Hs00902334_m1	
18S	Hs03928985_g1	

Supplementary Table 7: Outcomes of studies with co-supplementation or status of vitamin D with COVID-19 vaccination

S.No.	SARS-Co-2 Vaccine	Vitamin D Supplementation/ Serum Vitamin D levels	Outcomes	Reference
1.	BNT162b2	600 IU/day (14-16 weeks)	Increase in anti-SARS-CoV-2 IgG titers in participants.	[1]
2.	BNT162b2 and Sinovac vaccines	3200 IU/day (2 months)	Increase in anti-SARS-CoV-2 IgG titers in participants.	[2]
3.	BNT162b2	>50 nmol/L	Vitamin D levels showed a significant positive association with anti-spike IgG titers	[3]

4.	ChAdOx1 nCoV-19, BNT162b2	800 or 3200 IU/day (6 months)	Vitamin D supplementation did not influence the risk of breakthrough SARS-CoV-2 infection, anti-Spike titers (IgG, IgA, IgM), neutralizing antibody titers and IFN-γ concentrations in supernatants of S peptide-stimulated whole blood.	[4,5]
5	BNT162b2	>30 ng/mL	No effect of vitamin D levels on short-term response to a single dose of vaccine	[6]
6	BNT162b2	>30 ng/mL	No effect of vitamin D levels on anti-SARS-CoV-2 IgG titers	[7]
7	BNT162b2	≥30 ng/mL	No effect of vitamin D levels on anti-SARS-CoV-2 IgG levels	[8]

Supplementary References

1. Hawal Lateef Fateh, et al. "The Effect of Vit-D Supplementation on the Side Effect of BioNTech, Pfizer Vaccination and Immunoglobulin G Response against SARS-CoV-2 in the Individuals Tested Positive for COVID-19: A Randomized Control Trial." Clinical Nutrition Research, vol. 12, no. 4, 1 Jan. 2023, pp. 269–269, https://doi.org/10.7762/cnr.2023.12.4.269. Accessed 15 Apr. 2024.
2. Fatih Cesur, et al. Impact of Vitamin D3 Supplementation on COVID-19 Vaccine Response and Immunoglobulin G Antibodies in Deficient Women: A Randomized Controlled Trial. Vol. 41, no. 17, 1 Apr. 2023, pp. 2860–2867, www.ncbi.nlm.nih.gov/pmc/articles/PMC10040353/#:~:text=It%20is%20also%20stated%20that, https://doi.org/10.1016/j.vaccine.2023.03.046. Accessed 12 June 2023.
3. Piec, Isabelle, et al. "Age and Vitamin D Affect the Magnitude of the Antibody Response to the First Dose of the SARS-CoV-2 BNT162b2 Vaccine." Current Research in Translational Medicine, vol. 70, no. 3, 1 July 2022, pp. 103344–103344, https://doi.org/10.1016/j.retram.2022.103344. Accessed 15 Apr. 2024.
4. Jolliffe, David A, et al. "Influence of Vitamin D Supplementation on SARS-CoV-2 Vaccine Efficacy and Immunogenicity." MedRxiv (Cold Spring Harbor Laboratory), 17 July 2022, https://doi.org/10.1101/2022.07.15.22277678. Accessed 15 Apr. 2024.
5. Jolliffe, David A., et al. "Vitamin D Supplementation Does Not Influence SARS-CoV-2 Vaccine Efficacy or Immunogenicity: Sub-Studies Nested within the CORONAVIT Randomised Controlled Trial." Nutrients, vol. 14, no. 18, 1 Jan. 2022, p. 3821, www.mdpi.com/2072-6643/14/18/3821, https://doi.org/10.3390/nu14183821. Accessed 23 Feb. 2023.
6. Kofahi, Hassan M, et al. "Exploring the Effects of Vitamin D and Vitamin a Levels on the Response to COVID-19 Vaccine." Vaccines, vol. 11, no. 9, 21 Sept. 2023, pp. 1509–1509, https://doi.org/10.3390/vaccines11091509. Accessed 15 Apr. 2024.
7. Meyers, Eline, et al. "No Significant Association between 25-OH Vitamin D Status and SARS-CoV-2 Antibody Response after COVID-19 Vaccination in Nursing Home Residents and Staff." Vaccines, vol. 11, no. 8, 8 Aug. 2023, pp. 1343–1343, https://doi.org/10.3390/vaccines11081343. Accessed 17 Dec. 2023.
8. Chillon, Thilo Samson, et al. "Relationship between Vitamin D Status and Antibody Response to COVID-19 MRNA Vaccination in Healthy Adults." Biomedicines, vol. 9, no. 11, 18 Nov. 2021,

p. 1714, pubmed.ncbi.nlm.nih.gov/34829945/, https://doi.org/10.3390/biomedicines9111714. Accessed 22 Mar. 2022.
